# Assessing the Protective Metabolome Using Machine Learning in World Trade Center Particulate Exposed Firefighters at Risk for Lung Injury

**DOI:** 10.1038/s41598-019-48458-w

**Published:** 2019-09-03

**Authors:** George Crowley, Sophia Kwon, Dean F. Ostrofsky, Emily A. Clementi, Syed Hissam Haider, Erin J. Caraher, Rachel Lam, David E. St-Jules, Mengling Liu, David J. Prezant, Anna Nolan

**Affiliations:** 1 Department of Medicine, Division of Pulmonary, Critical Care and Sleep Medicine, New York University (NYU) School of Medicine, New York, USA; 2 Department of Population Health, Division of Health and Behavior, NYU School of Medicine, NY, New York, NY USA; 3 Department of Environmental Medicine, NYU School of Medicine, New York, NY USA; 4 Department of Population Health, Division of Biostatistics, NYU School of Medicine, NY, New York, NY USA; 5 Bureau of Health Services and Office of Medical Affairs, Fire Department of New York, Brooklyn, NY USA; 6 Pulmonary Medicine Division, Department of Medicine, Albert Einstein College of Medicine, Bronx, NY USA

**Keywords:** Biomarkers, Predictive markers

## Abstract

The metabolome of World Trade Center (WTC) particulate matter (PM) exposure has yet to be fully defined and may yield information that will further define bioactive pathways relevant to lung injury. A subset of Fire Department of New York firefighters demonstrated resistance to subsequent loss of lung function. We intend to characterize the metabolome of never smoking WTC-exposed firefighters, stratified by resistance to WTC-Lung Injury (WTC-LI) to determine metabolite pathways significant in subjects resistant to the loss of lung function. The global serum metabolome was determined in those resistant to WTC-LI and controls (n = 15 in each). Metabolites most important to class separation (top 5% by Random Forest (RF) of 594 qualified metabolites) included elevated amino acid and long-chain fatty acid metabolites, and reduced hexose monophosphate shunt metabolites in the resistant cohort. RF using the refined metabolic profile was able to classify cases and controls with an estimated success rate of 93.3%, and performed similarly upon cross-validation. Agglomerative hierarchical clustering identified potential influential pathways of resistance to the development of WTC-LI. These pathways represent potential therapeutic targets and warrant further research.

## Introduction

Rescue workers of the Fire Department of New York (FDNY) exposed to World Trade Center particulate matter (WTC-PM) had heterogeneity in lung function outcomes^1–6^. Our group has identified metabolically active biomarkers associated with WTC-Lung Injury (WTC-LI) resistance; however, little is known about the bioactive metabolites relevant after WTC exposure in this population^3,7,8^. Metabolomic profiling provides comprehensive quantification of small organic molecules, yielding a single-time-point snapshot^9^. In a non-invasive functional genetics approach to describing molecular complexity, the metabolome’s assessment is a proximal link to disease phenotype^10^.

However, similar to other high-throughput ‘omic platforms, there are two main challenges to data analysis: determining which variables are relevant to our endpoint, and visualizing this data to derive insight^11^. The classical biomarker methodology of significance-testing followed by post-hoc correction and multivariate regressions falls short due to its inability to analyze variable interaction early on in the workflow. This shortfall leads to the potential of false negatives and false positives. For this reason, we utilize a machine learning approach, which has become popular in recent years, is highly relevant in its application to metabolomic classification, feature selection, and feature projection, and can classify subjects based on non-trivial structures and variable interactions inherent in the data, providing a better overview of the relevant metabolites. We rely on Random Forests (RF) for this initial selection of a refined metabolite profile maximally relevant to class differentiation. Briefly, RF is a non-parametric, ensemble-based classifier that rarely overfits, is not sensitive to variable scale, and works well on datasets with sizes similar to ours^12^. RF records an unbiased measure of each variable’s importance to classification success rate, called mean-decrease-accuracy (MDA), and we can select the highest-MDA metabolites for our refined profile.

To further our data interpretation, dimension reduction techniques were employed. Specifically, principal components analysis (PCA) of the refined profile provided a low-dimensional view that captures maximal variance. We uncovered structure in the refined profile of the metabolome through agglomerative, hierarchical clustering of the data and correlation matrices. We hypothesize that implementing high-dimensional data analysis and dimension reduction techniques on the metabolomic fingerprints of WTC-PM exposure in the serum of firefighters will further refine biologically relevant pathways of resistance to WTC-LI.

## Results

### Demographics

**Figure 1:**
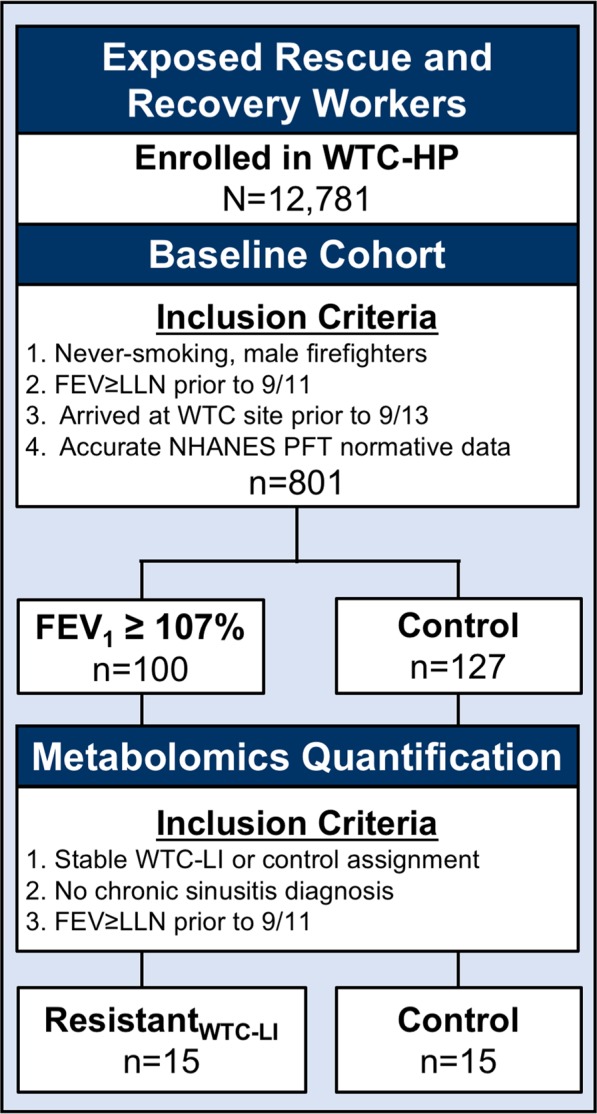
Study Design.

Derivation of cases and controls as described in our methods are shown in Fig. 1. Overall, resistant_WTC-LI_ with metabolome assessed did not differ from their parent cohort in spirometry, body mass index (BMI), age at exposure at the WTC site (age on 9/11), exposure intensity, lipid profiles (including triglycerides, high- and low-density lipoproteins), blood pressure, heart rate, leukocyte differential percents (neutrophil, lymphocyte, monocyte, basophil, and eosinophil), or serum sodium, chloride, potassium, glucose, uric acid, total protein, calcium, phosphorous, iron, CO_2_, albumin, blood urea nitrogen (BUN), creatinine, albumin/creatinine, or BUN/creatinine. Controls with metabolome assessed and their parent cohort did not differ in lipids, leukocyte differential percents, or serum sodium, chloride, potassium, glucose, uric acid, total protein, BUN, creatinine, or BUN/creatinine, Table 1.

**Table 1:** Clinical Characterization of Parent Cohort and Metabolomics Subcohort.

Measure		Parent Cohort		Metabolomics Subcohort	
		Controls n = 127	Resistant_WTC-LI_ n = 100	Controls n = 15	Resistant_WTC-LI_ n = 15
PFT at SPE	FEV_1, % Pred_^a,b^	93 (85–99)	113 (109–118)	92 (90–98)	118 (111–125)
	FVC_% Pred_^a,b^	96 (89–103)	110 (106–116)	97 (95–100)	112 (107–121)
	FEV_1_/FVC^a,b^	76 (73–80)	82 (79–84)	75 (71–82)	82 (79–86)
BMI (kg/m^2^)	WTC-HP Entry^a,c^	28 (26–30)	27 (26–29)	26 (25–27)	27 (26–28)
	SPE^a,c^	29 (27–31)	28 (26–30)	26 (24–28)	28 (25–30)
	Age on 9/11 (years)	41 (37–45)	42 (37–46)	42 (38–46)	42 (38–46)
Exposure n(%)	Low	13 (10%)	9 (9%)	1 (7%)	2 (13%)
	Intermediate	85 (67%)	71 (71%)	11 (73%)	8 (53%)
	High	29 (23%)	20 (20%)	3 (20%)	5 (33%)
Duration (months)^d^		3 (1–5)	3 (1–6)	2 (1–5)	2 (1–3)
Lipids (mg/dL)	Triglycerides^a^	164 (98–238)	124 (94–191)	126 (99–237)	128 (107–195)
	HDL	47 (40–55)	47 (40–54)	48 (45–57)	50 (43–61)
	LDL	131 (104–157)	128 (107–153)	134 (100–144)	142 (108–157)
Heart Rate (beats/min)^c^		72 (66–76)	72 (66–76)	66 (64–70)	72 (64–74)
BP (mmHg)	Systolic^c^	114 (108–124)	118 (110–122)	110 (100–112)	112 (108–120)
	Diastolic^c^	70 (70–80)	72 (70–80)	70 (60–72)	70 (66–74)

Values shown as n(%) or Median (IQR). Significance by Mann-Whitney *U* observed between: a—127 vs. 100; b—15 vs. 15; c—127 vs. 15 controls. Data available for: d—14 subcohort resistant_WTC-LI_.

Resistant_WTC-LI_ had decreased serum levels of sodium compared to their parent cohort. Controls had significantly lower BMI at entry into WTC-Health Program (WTC-HP) and subspecialty pulmonary examination (SPE), and significantly higher heart rate and blood pressure than their parent cohort; however, the controls with metabolome assessed have similar BMIs at WTC-HP and SPE compared to resistant_WTC-LI_ with metabolome assessed, and the same trend remains true for heart rate and blood pressure, Table 1.

As expected, controls that had their metabolome assessed had lower baseline lung function at SPE compared to resistant_WTC-LI_, as was the case with their respective parent cohorts. The BMI at WTC-HP entry and SPE of the control parent cohort was significantly increased compared to the resistant_WTC-LI_ parent cohort, Table 1. Similarly, controls that had their metabolome quantified had higher serum levels of uric acid than resistant_WTC-LI_ with metabolome assessed, but no differences in sodium, chloride, potassium, glucose, total protein, calcium, phosphorous, iron, CO_2_, albumin, BUN, creatinine, albumin/creatinine, BUN/creatinine, clinically available lipids, blood pressure, heart rate, or leukocyte subtype percents.

### Metabolomics

**Figure 2:**
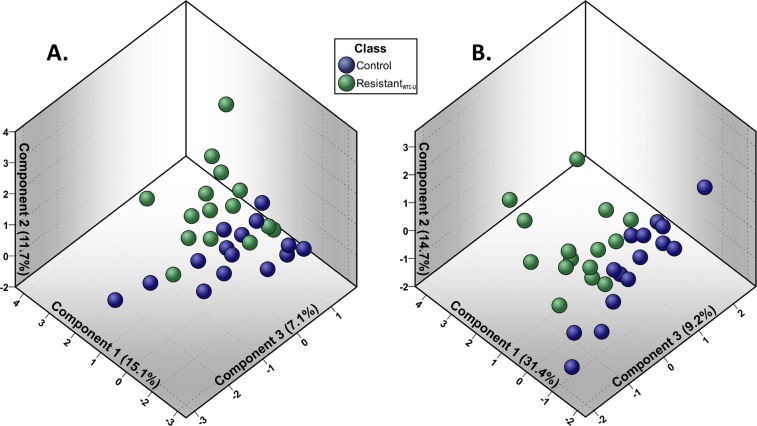
Demonstration of Model Optimization: PCA Scores Plot. (**A**) PCA of the qualified profile reveals heterogeneity in the data. (**B**) PCA of the refined profile demonstrates improved class separation produced by the refined profile compared to initial PCA (panel A).

**Figure 3:**
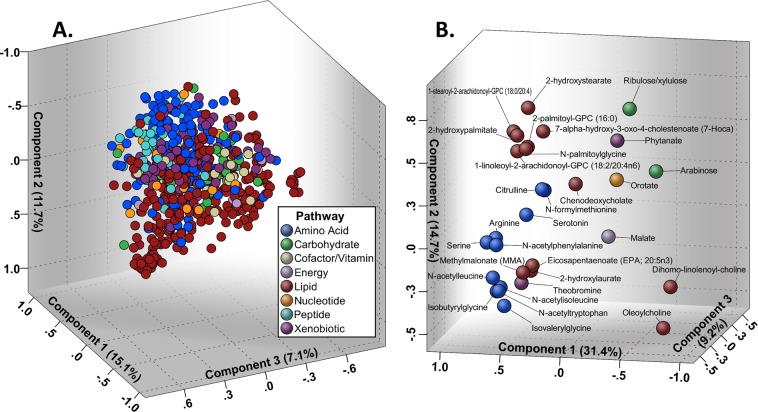
(**A**) Qualified Profile PCA Loading Weights Plot. Loading weights plot of PCA of the qualified profile shows ill-defined metabolite clustering. (**B**) Refined Profile PCA Loading Weights Plot was used to derive insight into possible associations of biomarkers.

**Figure 4:**
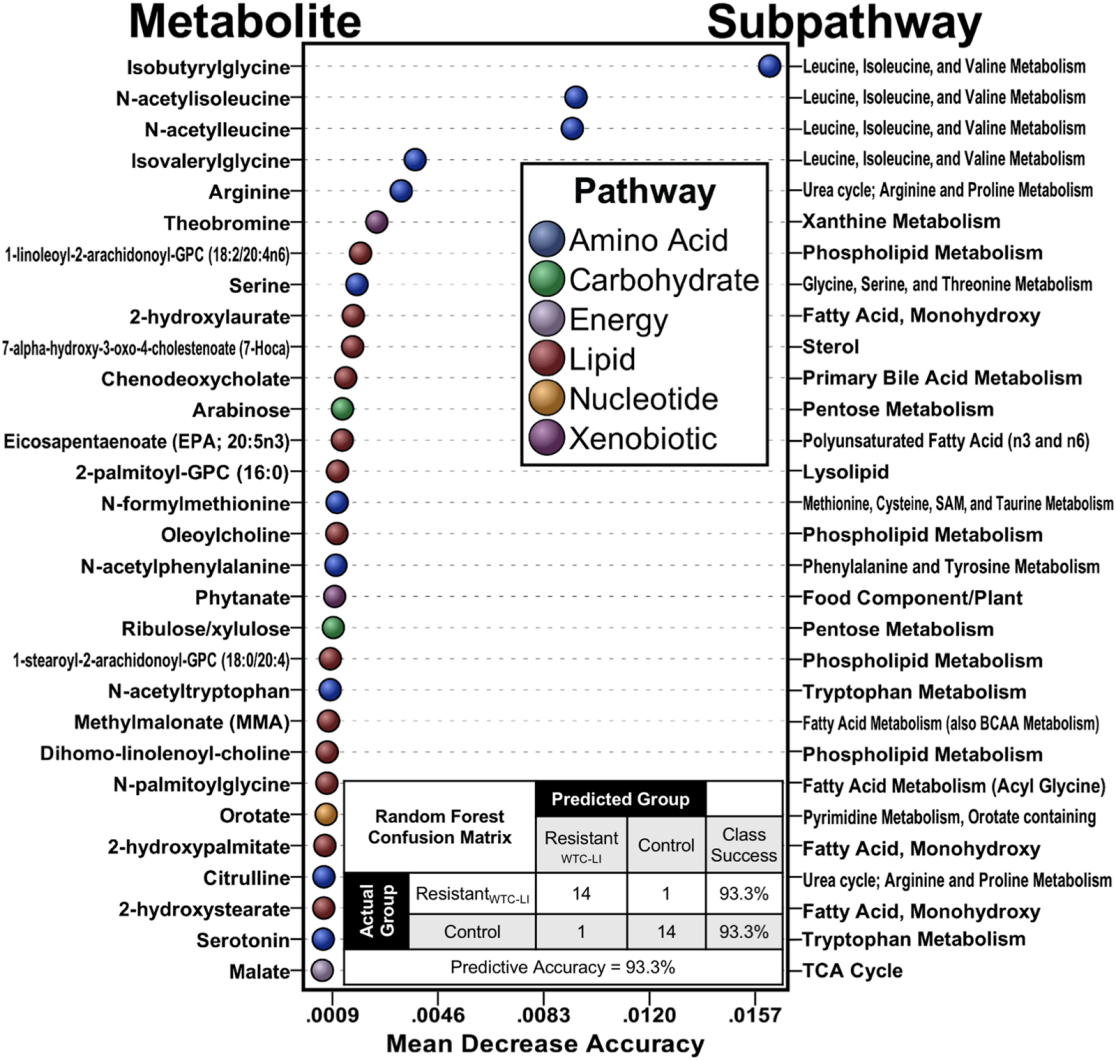
Random Forests Variable Importance in Projection. RF variable importance in projection is measured by mean decrease accuracy; the top 5% of metabolites important to class separation are shown. The confusion matrix shows classification accuracy of the refined profile.

We have included raw area counts, mass, retention index, and the platform on which each metabolite was identified in a Supplementary File, Data S3. The raw area counts correspond to area under the curve measurements for each metabolite, thus serving as measurements of abundance level. Initially, 759 metabolites were identified. Of these, 594 qualified for further analysis, Table S1. Preliminary PCA of this set of metabolites bore relatively weak class separation based on metabolite expression levels in the qualified profile of resistant_WTC-LI_ and controls, Fig. 2A, and no clear metabolite clustering patterns, Fig. 3A. Metabolites that met qualification criteria were included in the first RF, which ranked metabolite importance to class separation, and yielded the refined metabolite profile, Fig. 4. In the second run of RF, only the refined metabolite profile was analyzed, achieving a 6.7% out-of-bag estimated error rate (estimated accuracy of 93.3%), Fig. 4.

A second pass of PCA, including only metabolites in the refined panel captured 76.7% of the variance in 7 components as determined by scree plot inspection, Fig. S1B. Here, there was marked improvement in class clustering compared to preliminary PCA, Fig. 2B. Additional, 2-dimensional PCA scores plots are available, Fig. S2. There were also localized clusters in the PCA loading weights plot, Fig. 3B.

**Figure 5:**
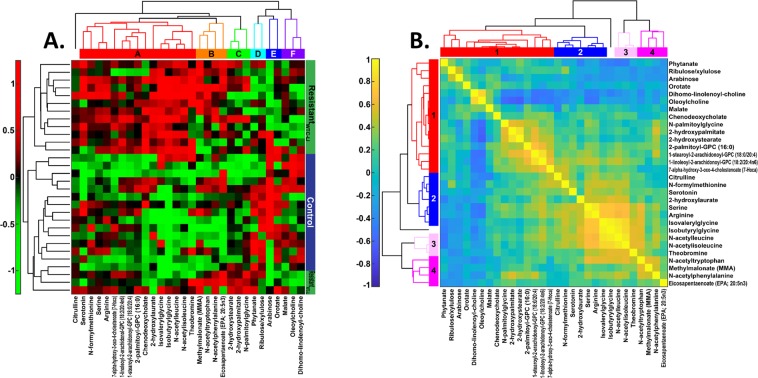
Agglomerative, Hierarchical Clustering. (**A**) Data Matrix. Clustering of the data matrix identified 6 clusters of metabolites (A-F) and separated resistant_WTC-LI_ from controls. (**B**) Correlation Matrix. Clustering of the correlation matrix reveals 4 clusters of metabolites (1-4) and intercluster correlations.

Agglomerative hierarchical clustering was then performed on the data and correlation matrices of the refined profile, Fig. 5A,B. Linkage thresholds determined by inspection of the dendrograms were used to highlight clusters of metabolites that may reflect mechanistic relations. For the data matrix, a linkage threshold of 0.73 was used to identify 6 distinct clusters of metabolites (A-F), Fig. 5A. A linkage threshold of 0.60 highlighted 4 clusters (1-4) in the correlation matrix, Fig. 5B.

Cluster-A consisted largely of amino acids and their metabolites, including acyl/acetylated, branched-chain amino acids and those of the urea cycle. Meanwhile, cluster-B was more diverse, containing methylmalonate, some n-acetyl amino acids, and eicosapentanoate (EPA; 20:5n3). A total of three fatty acids (FA) comprised cluster-C, which was similar to clusters-A and -B. A second, relatively removed branch contained the remaining three clusters-D-F. Of these, clusters-D and -E were characterized by sugar intermediates of the pentose phosphate pathway, and cluster-F by cytosolic intermediates. Clusters 1-4 identified by the correlation matrix resembled clusters-A-F in composition and structure. The intra- and intercluster correlations in Fig. 5B can be used to understand how metabolites and clusters interact, and corroborate patterns in Fig. 5A. As expected, strong correlations were found among related metabolites, Fig. 5B.

## Discussion

WTC-PM-exposed firefighters’ serum banked within 6 months of 9/11 is a metabolically diverse environment. While the metabolome describes multifaceted interactions amongst various parent cells, our data suggests, as expected, that the metabolites that are most bioactive and associated with WTC-LI are amino acids and lipids. In studies of broader categories of obstructive airways disease, some of these metabolites have been found to be relevant. As hypothesized, under clustering analysis, these previously observed metabolites organized according to known mechanisms. There were, however clusters containing novel metabolites that may reflect as yet defined signaling cascades.

Resistant_WTC-LI_ had increased concentrations of a variety of amino acid-related compounds, including numerous acetylated/acylated metabolites of aliphatic, aromatic, and branched-chain amino acids^13,14^. Specifically, these leucine, isoleucine, and valine metabolites included those with the top four MDA scores—isobutyrylglycine, n-acetylisoleucine, n-acetylleucine, and isovalerylglycine. The biologic function of these molecules in relation to lung injury are unclear, and their relative accumulation could indicate more acetyl/acyl donors or higher protein intakes among firefighters without lung injury^15^. The increased concentrations of urea cycle intermediates, citrulline and arginine, support the latter hypothesis^16^. Generalized increases of these proteins are reflected by the metabolites in cluster-A, Fig. 5A. Although adequate protein is important for resistance and response to damage from PM exposure, it seems unlikely that protein deficiency was a major issue among active firefighters; however, differences in protein intakes could result from other characteristics of firefighters that were related to lung injury status^17,18^.

**Figure 6:**
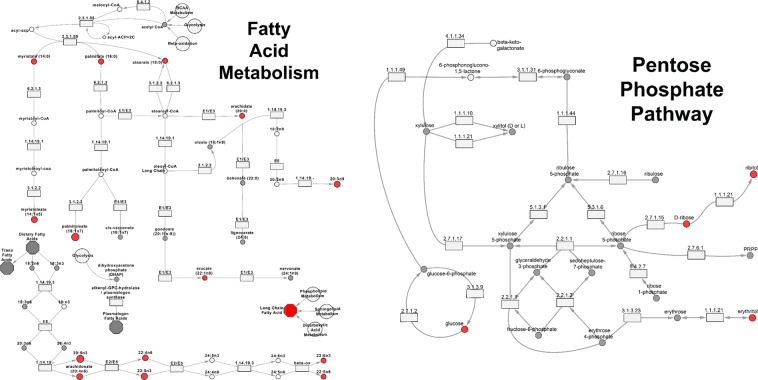
Pathway Schematics. Pathway schematics of fatty acid metabolism and the hexose monophosphate shunt. Node size correlates to fold change, red indicates fold change >1, resistant_WTC-LI_/control.

Numerous lipid metabolites were also associated with protection from lung injury. In particular, eicosapentaenoic acid (EPA, 20:5n-3), a long-chain n-3 FA, was increased in firefighters without lung injury. EPA is a precursor for local signaling molecules, notably eicosanoids, which are important for mediating inflammatory and immune response to injury, Fig. 6. Eicosanoids derived from EPA are widely regarded as being responsible for the anti-inflammatory effects of n-3 FAs, and may have helped reduce the lung damage caused by exposure to dust and smoke^19^. This explanation is in line with our prior observation of EPA metabolites, specifically docosahexanoate derivatives, as protective against WTC-LI^20^. We have also previously identified high levels of arachidonate, an eicosanoid and n-6 FA derivative^20^. The metabolism of n-3 and -6 FA derivatives induce anti- and pro-inflammatory responses, respectively^21,22^. In this context, the n-6/n-3 FA ratio determines the nature of the response, can be regulated through unsaturated fat intake, and may serve as preventive or regulatory treatment for WTC-LI^22–25^. Interestingly, the n-3 FA we observed, EPA, was negatively correlated with members of clusters D-F, which contain intermediates of the hexose monophosphate shunt (HMS; also called the pentose phosphate pathway).

Lastly, intermediates of the HMS were reduced in resistant_WTC-LI_. The HMS runs parallel to glycolysis in cells, generates ribose-5-phosphate, and reduced nicotinamide adenine dinucleotide phosphate (NADPH), Fig. 6. These components are needed for synthesizing nucleotides and lipids, and regenerating the antioxidant glutathione (GSH). It is unclear whether low HMS intermediate concentrations indicate high or low pathway activation. One possible explanation for this finding is that lung injury increases the demand for nucleotides for reparative cell division and antioxidants for removing free radicals, causing an increase in HMS intermediates among those with lung injury^16^. An increase in free radicals may be due to the heavy metals, asbestos, and silica in WTC-PM, which can induce oxidization of GSH^26–28^ and turnover of phosphatidylcholines to activate pro- and anti-inflammatory pathways^21,22^.

There are several limitations to this study. This analysis is a single-time-point assessment of the metabolome. Therefore, we attempt to minimize false discoveries through the use of machine learning algorithms that are robust to noise, and we emphasize the exploratory nature of this analysis. A discussion of the strengths and limitations of the methods of analysis is therefore warranted. The RF, PCA, and clustering algorithms allow us to efficiently identify inherent patterns and intermetabolite relations within the data. That being said, our analyses and data characteristics preclude us from establishing causation; we aim to elucidate pathways of interest to be examined in a larger cohort, *in-vivo*, and *in-vitro*. High variable-to-noise ratios can typically result in fitting models to noise. While the RF model built in this study is specific to this dataset, RF uses a repeated random subsampling procedure (also known as bootstrap-aggregating or “bagging”) that averts, to the extent possible, the issues raised by the sample size. We use out-of-bag classification as an internal cross-validation method, and plan to externally validate our findings in a larger cohort.

Additional attempts to control the integrity of the dataset include efforts made in case selection; the groups are, for the most part, similar, save for select metabolic biomarkers. While we observe BMI differences at baseline, potential related effects were controlled by the use of lung function percent predicted values as case definition. Additionally, this analysis is limited to never-smokers; the metabolome of the ever-smoking, WTC-exposed population remains to be quantified. Drug therapy may also influence the metabolome. The unknown prevalence of medication use in the current study emphasizes the need for larger-scale validation, but a small group of patients with drug therapy are unlikely to sway the present analysis.

The control population in this manuscript was considered in our prior publication that assessed active pathways in patients that developed WTC-LI^20^. While the control population is shared by both papers, the case definition of interest in this paper focuses on resistance to WTC-LI (as opposed to susceptibility in the prior publication). Thus, the metabolite profiles differ; the qualified profiles were substantively different, and each refined profile consists of distinct metabolites. This indicates that the molecular pathways involved in WTC-LI resistance may be independent of those involved in WTC-LI pathogenesis. Therefore, the works presented are each biologically significant in their own right, and contribute new and unique information regarding biologically plausible cascades.

Pathogenesis in the lung following PM exposure is a complex process involving a wide variety of pro- and anti-inflammatory signaling cascades. Large-scale omics research such as this is important to describing these processes. Using our approach, we may then be able to isolate the most active set of pathways involved in disease and set these as the target for tailored pharmacotherapy. To this end, we highlight several metabolically active pathways, including branched-chain and other amino acid metabolism, essential n-3 FAs, and the HMS. These pathways may represent modifiable risk factors that can be targeted to ameliorate disease state. Further research includes the validation of the pathways identified in this paper in a larger cohort, and a dietary intervention to improve lung function in firefighters by targeting some of these pathways.

## Methods

### Study design

Both cases and controls were sampled from symptomatic individuals referred for SPE from 10/1/2001 to 3/10/2008, as previously described^1,7,29^. This study’s parent cohort included subjects resistant to WTC-LI (resistant_WTC-LI_; n = 100) and was defined as having an FEV_1,%Pred_ within one standard deviation of the highest FEV_1,%Pred_ at SPE, while controls (n = 127) were randomly sampled from tertiles of BMI and FEV_1,%pred_ at WTC-HP entry^1,7,29^.

Subjects that underwent untargeted metabolomics were identified using the following inclusion criteria: having a stable resistant_WTC-LI_/control assignment (including the most recent spirometric measures on their annual health physical), and not having a diagnosis of chronic rhinosinusitis. Based on these criteria and availability of subjects, (n = 15) resistant_WTC-LI_ at a 1:1 ratio with randomly selected controls (n = 15) were identified, Fig. 1 ^1,29–31^.

Demographics were obtained from the WTC-HP. WTC exposure intensity was categorized by the FDNY-WTC Exposure Intensity Index according to first arrival time at the WTC site^5,32,33^. High exposure subjects are those that arrived during the morning of September 11, 2001. Intermediate exposure subjects arrived in the afternoon of September 11, 2001. Low exposure subjects arrived on September 12, 2001. Duration is the number of months spent performing rescue and recovery efforts at the WTC site^31^. BMI was measured at HP enrollment and SPE. All subjects at the time of their enrollment signed informed consent and agreed to the analysis of their data and samples. Overall, the consent and the experimental protocol were approved by the Institutional Review Boards at Montefiore Medical Center (#07-09-320) and New York University (#16-01412). Furthermore, all experiments conformed to the relevant regulatory standards.

### Metabolomics

Serum was collected and stored within 200 days after 9/11/2001^1,7,29,34,35^. Serum aliquots dedicated for metabolomics were stored at −80 °C until processed and quantified using the automated MicroLab STAR® (Hamilton)^20^.

Proteins were methanol precipitated and the extract was aliquoted into 5 fractions, each of which was used to identify different subtypes of metabolites: 2 for reverse phase (*RP*)*/Ultrahigh Performance Liquid Chromatography-Tandem Mass Spectroscopy* (*UPLC-MS/MS*) *methods with positive ion mode electrospray ionization* (*ESI*), one for *RP/UPLC-MS/MS with negative ion mode ESI*, one for *HILIC/UPLC-MS/MS with negative ion mode ESI*, and one backup sample was maintained. All methods utilized a Waters ACQUITY ultra-performance liquid chromatography (UPLC) and a Thermo Scientific Q-Exactive high resolution/accurate mass spectrometer interfaced with a heated electrospray ionization (HESI-II) source and Orbitrap mass analyzer operated at 35,000 mass resolution. The sample extract was dried, then reconstituted in solvents compatible with each of the four methods. Each reconstitution solvent contained a series of standards at fixed concentrations to ensure injection and chromatographic consistency.

The four aliquots were analyzed as follows: 1) using acidic positive ion conditions, chromatographically optimized for more hydrophilic compounds. In this method, the extract was gradient eluted from a C18 column (Waters UPLC BEH C18-2.1 × 100 mm, 1.7 µm) using water and methanol, containing 0.05% perfluoropentanoic acid (PFPA) and 0.1% formic acid; 2) using acidic positive ion conditions, chromatographically optimized for more hydrophobic compounds. In this method, the extract was gradient eluted from the same aforementioned C18 column using methanol, acetonitrile, water, 0.05% PFPA, and 0.01% formic acid, and was operated at an overall higher organic content; 3) using basic negative ion optimized conditions using a separate, dedicated C18 column. The basic extracts were gradient eluted from the column using methanol and water, however, with 6.5 mM ammonium bicarbonate at pH 8; 4) via negative ionization following elution from a HILIC column (Waters UPLC BEH Amide 2.1 × 150 mm, 1.7 µm) using a gradient consisting of water and acetonitrile with 10 mM ammonium formate, pH 10.8. The MS analysis alternated between MS and data-dependent, n^th^-order MS scans using dynamic exclusion. The scan range varied between methods but covered 70-1,000 mass-to-charge.

Several types of controls were analyzed in concert with the experimental samples: a pooled matrix sample generated by taking a small volume of each experimental sample (or alternatively, use of a pool of well-characterized human plasma) served as a technical replicate throughout the data set; extracted water samples served as process blanks; and a cocktail of quality control standards that were carefully chosen not to interfere with the measurement of endogenous compounds were spiked into every analyzed sample, allowed instrument performance monitoring, and aided chromatographic alignment.

The bioinformatics system consisted of four major components, the Laboratory Information Management System (LIMS), the data extraction and peak-identification software, data processing tools for quality control and compound identification, and a collection of information interpretation and visualization tools. The hardware and software foundations for these informatics components were the local-area network backbone, and a database server running Oracle 10.2.0.1 Enterprise Edition. The scope of the Metabolon LIMS system encompasses sample accessioning, sample preparation and instrumental analysis, and reporting and advanced data analysis. All of the subsequent software systems are grounded in the LIMS data structures. It has been modified to leverage and interface with the in-house information extraction and data visualization systems, as well as third party instrumentation and data analysis software.

In-house peak detection and integration software produced data output including mass-to-charge ratios, retention indices, and area under the curve values. Biochemical identification was performed by matching compounds to a library of mass, retention index, and spectral data as previously described^20,36–39^. Metabolite identification was based on three criteria: retention index within a narrow retention index window of the proposed identification, accurate mass match to the library +/− 10 parts per million, and the MS/MS forward and reverse scores between the experimental data and authentic standards. The MS/MS scores are based on a comparison of the ions present in the experimental spectrum to the ions present in the library spectrum. The library entries were built using the exact methods used in the current study to assure the greatest comparability between the experimental values and the library values. Further quality control and curation achieved consistent identification of valid metabolites, and removal of system artifact, mis-assignments, and noise. Every metabolite assignment was manually approved, and the assignment was confirmed by a second reviewer. This renders the majority of the identifications as Metabolomics Standards Initiative level 1 when using the classification of Sumner *et al*. Further details about this process are available^40–42^. Missing data was imputed with the minimum observed level per metabolite^20,43^.

### Database management and statistics

The database was maintained and handled in SPSS-23 (IBM). Continuous and ordinal variables were expressed as median and inter-quartile range. Mann-Whitney U-test was used to compare continuous and ordinal data. Count and proportions were used to summarize categorical data and Pearson-𝜒^2^ was used for comparison.

### Feature selection—machine learning

Metabolite data pre-processing isolated metabolites that were observed in ≥80% of subjects per group with ≥15% relative standard deviation^20,40^. Using RF (randomForest Package R-3.4.3, R-Project) we identified our qualified metabolite profile and analyzed it in an iterative process of variable selection and assessment of classification accuracy of selected variables. The default number of metabolites were assessed at each node, and 10 replicates of the model were trained on the same data and assessed via estimated out-of-bag error rate to verify stability. The first round of RF output 10 unique-but-similarly-performing models consisting of 10^6^ trees to ensure stability. Metabolites with MDA scores within the highest 5% of scores of the best-performing model (as measured by estimated out-of-bag error rate) comprised the refined metabolite profile. In the second round of RF, models consisting of 10^3^ trees were trained on the refined profile^12^.

### Hyperparameter tuning

Due to the exploratory nature of this study, our goal was to identify the most discriminative metabolites in our study cohort. Therefore, the size of the forests was determined to discover the set of most discriminative metabolites by maximizing refined profile membership and rank consistency. This was accomplished by assessing these factors within the prospective refined profiles (the top 5% of metabolites by mean decrease accuracy) of many random forests; the hyperparameter tuning was performed by searching half-magnitude steps of the size of the forest, ranging from 10^0^ to 10^6^ trees. In each stratum, 10 random forests were grown, the pairwise hamming distances and number of unique elements in the prospective refined profiles were calculated, and reported as an average per forest size. The final qualified random forests model of 10^6^ trees minimized variations in the average hamming distance and prospective refined profile membership. We present the tuning process of the qualified random forests in Fig. S3A.

Then, we aimed to assess the classification utility of the refined metabolite profile. We developed a procedure in which 10 replicate random forest models were grown using 5-fold cross-validation at each forest size, again scanning the range from 10^0^ to 10^6^ in half-magnitude steps. The classification accuracy per forest was measured as the average accuracy of all its folds. We then calculated the mean and standard deviation of the classification accuracies per forest size, and determined the optimal forest size as that which minimized the spread of classification accuracy across replicate models and maximized classification accuracy. We present the tuning process of the refined profile random forests in Fig. S3B.

### Feature projection

PCA (SPSS 23, IBM) was used to obtain a low-dimensional representation of the qualified and refined profiles. PCA was performed on the qualified profile, then on the refined profile. This process allowed visualization of the increasing resistant_WTC-LI_/control separation as metabolites present in the qualified profile but not relevant to the clinical endpoint were removed from consideration. PCA scores are plotted to estimate where subjects reside in higher-dimensional space; loading weights are plotted to indicate the influence of the metabolites on the components, and can reveal intermetabolite associations by correlation. The variance captured with PCA is calculated as the sum of the variance captured by the principal components identified in the scree plot.

Additionally, unsupervised, agglomerative, hierarchical clustering was carried out on data and correlation matrices of the refined metabolite profile. For both matrices, the average linkage method was used. For the data matrix, the spearman distance was used as a similarity measure on log-transformed data to control for outliers and scale differences. For the correlation matrix, the correlation distance was used as a similarity measure (MATLAB, MathWorks).

## Supplementary information

Supplementary Information

Data S3
