# Supplementary material for: Assessing the Protective Metabolome Using Machine Learning in World Trade Center Particulate Exposed Firefighters at Risk for Lung Injury
Source: Sci Rep. 2019 Sep 3;9:11939. doi: 10.1038/s41598-019-48458-w (PMC6722247; doi:10.1038/s41598-019-48458-w)
Supplement: Supplementary file 1 — Supplementary Information [file 41598_2019_48458_MOESM1_ESM.pdf]

Assessing the Protective Metabolome Using Machine Learning in World Trade Center Particulate Exposed Firefighters at Risk for Lung Injury

George Crowley[1], Sophia Kwon[1], Dean F. Ostrofsky[1], Emily A. Clementi[1], Syed Hissam Haider[1], Erin J. Caraher[1], Rachel Lam[1], David E. St-Jules[2], Mengling Liu[3, 4], David J. Prezant[5, 6], Anna Nolan*[,1, 3, 5]

Supplementary Information

Supplementary Figure Legends

Figure S1. Scree Plots. Scree plots guide determination of the number of components to retain in principal components analysis of (**A**) the qualified metabolite profile and (**B**) the refined metabolite profile.

Figure S2. 2-dimensional PCA Scores Plots. 2-dimensional PCA scores plots provide a streamlined perspective of principal components analysis of (**A**) the qualified metabolite profile and (**B**) the refined metabolite profile.

Figure S3. Random Forests Hyperparameter Tuning. A. Qualified profile random forests were tuned to consistently identify the most discriminative metabolites by minimizing hamming distance and maximizing prospective refined profile membership consistency between replicate models of a given forest size. **B.** Refined profile random forests were tuned to maximize classification accuracy of replicate models of given forest size trained using 5-fold cross-validation.

Supplementary Materials

Table S1. Fold Changes of Metabolites in Qualified Profile.

Table S2. Fold Changes of Metabolites in Refined Profile.

Data S3. Raw Metabolite Data.

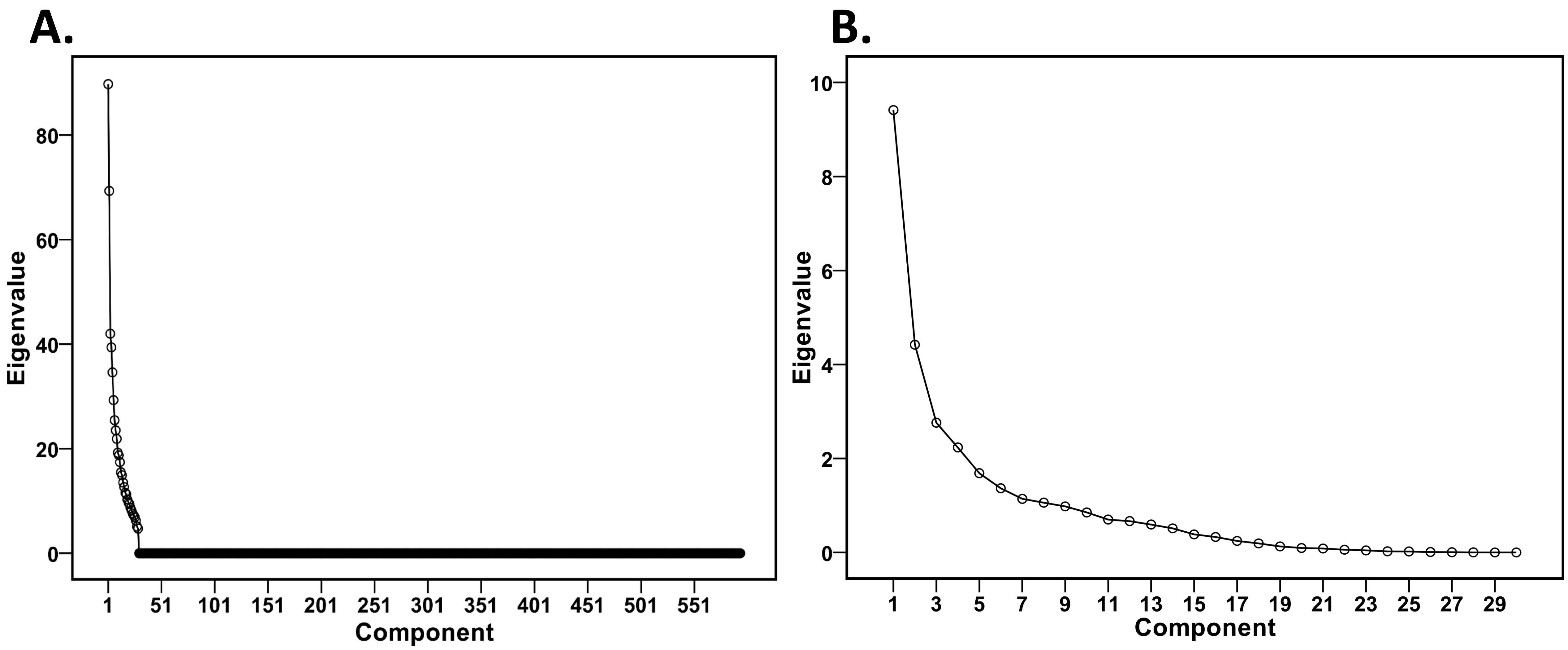

Figure S1. Scree Plots.

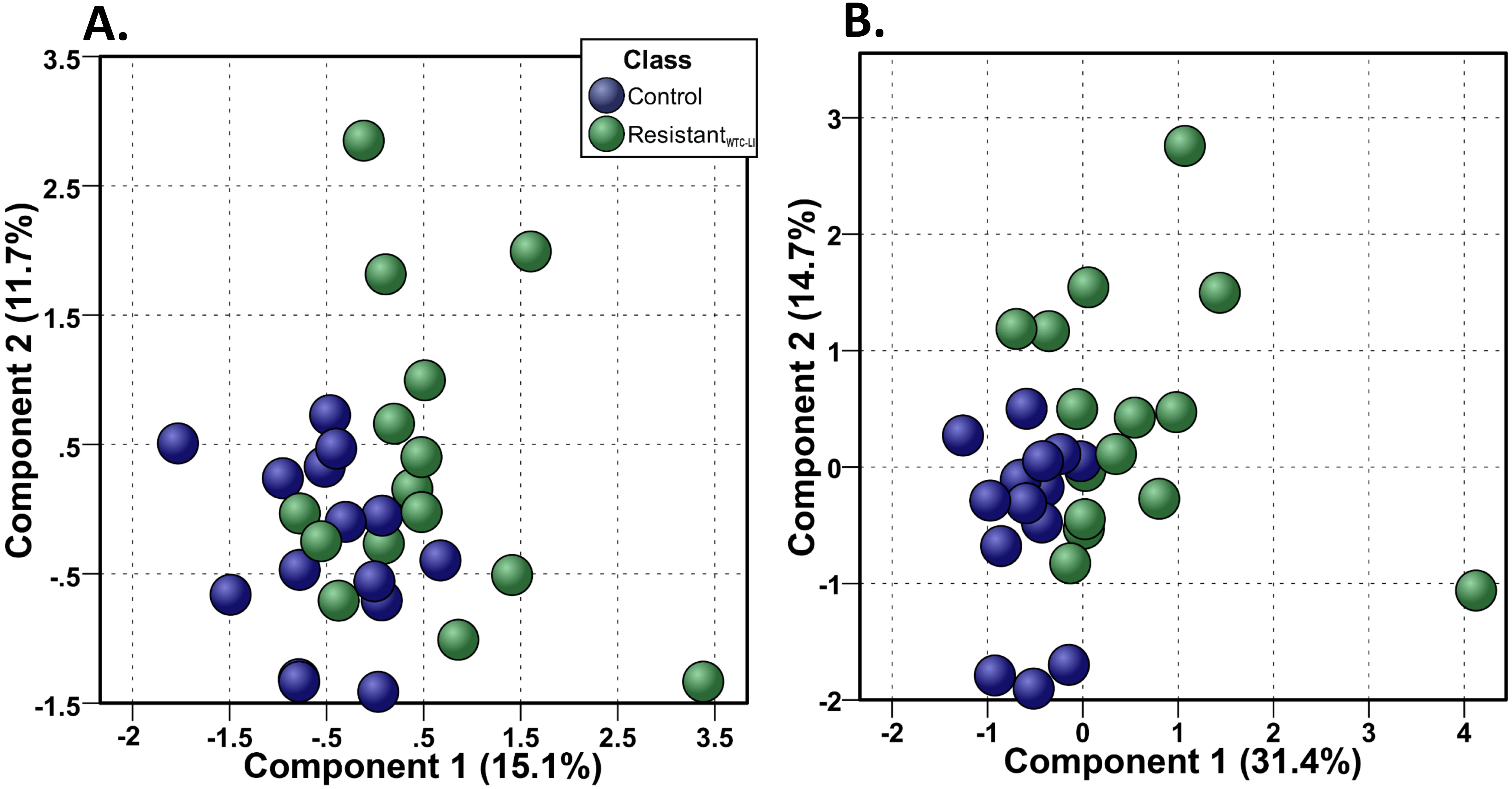

Figure S2. 2-dimensional PCA Scores Plots.

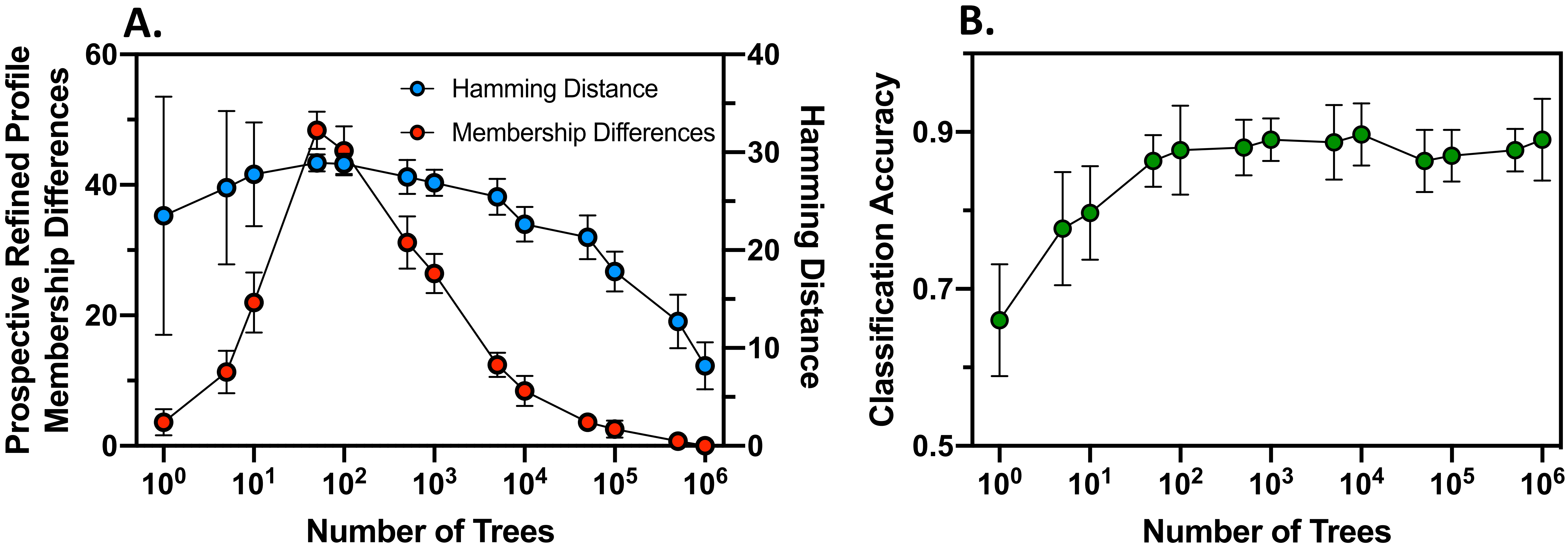

Figure S3. Random Forests Hyperparameter Tuning.

Table S1. Fold Changes of Metabolites in Qualified Profile

Fold Change: Resistant$_{WTC-LI}$/Control

Legend	
(green)	<1
(red)	>1

Super Pathway	Sub Pathway	Metabolite	Comp ID	KEGG	HMDB	PubChem	Resistant$_{WTC-LI}$ / Control	CAS	Retention Index	Mass
Amino Acid	Glycine, Serine and Threonine Metabolism	glycine	58	C00037	HMDB00123	750		56-40-6	1375	76.04
		N-acetylglycine	27710		HMDB00532	10972		543-24-8	731	116.04
		sarcosine	1516	C00213	HMDB00271	1088		107-97-1	1280	90.05
		dimethylglycine	5086	C01026	HMDB00092	673		1118-68-9	1104	104.07
		betaine	3141	C00719	HMDB00043	247		107-43-7	1064	118.09
		serine	1648	C00065	HMDB00187	5951		56-45-1	1239	106.05
		N-acetylserine	37076		HMDB02931	65249		97-14-3	2000	146.05
		threonine	1284	C00188	HMDB00167	6288		72-19-5	1514	120.07
		N-acetylthreonine	33939			152204		17093-74-2	821.6	160.06
		allo-threonine	15142	C05519	HMDB04041	99289		28954-12-3	2511.1	118.05
	Alanine and Aspartate Metabolism	alanine	1126	C00041	HMDB00161	5950		56-41-7	1700	90.05
		N-acetylalanine	1585	C02847	HMDB00766	88064		97-69-8	861.2	130.05
		aspartate	443	C00049	HMDB00191	5960		56-84-8	1165	134.04
		asparagine	512	C00152	HMDB00168	6267		70-47-3	1225	133.06
		N-acetylasparagine	33942		HMDB06028	99715		4033-40-3	785	175.07
		N-acetylaspartate (NAA)	22185	C01042	HMDB00812	65065		997-55-7;997-55-7	880	176.06
	Glutamate Metabolism	glutamate	57	C00025	HMDB00148	611		56-86-0	1500	148.06
		glutamine	53	C00064	HMDB00641	5961		56-85-9	1291	147.08
		N-acetylglutamate	15720	C00624	HMDB01138	70914		8/3/5817	1050	190.07
		N-acetylglutamine	33943	C02716	HMDB06029	182230		2490-97-3	2140	187.07
		N-acetyl-aspartyl-glutamate (NAAG)	35665	C12270	HMDB01067	5255		3106-85-2	1035	305.1
		glutamate, gamma-methyl ester	33487			68662		1499-55-4	2170	162.08

		pyroglutamine*	46225			134508		2353-44-8	1900	129.07
		beta-citrylglutamate	54923					73590-26-8	900	322.08
	Histidine Metabolism	histidine	59	C00135	HMDB00177	6274		5934-29-2	755.9	154.06
		N-acetylhistidine	33946	C02997	HMDB32055	75619		39145-52-3	2088	196.07
		1-methylhistidine	30460	C01152	HMDB00001	92105		332-80-9	2755	170.09
		3-methylhistidine	15677	C01152	HMDB00479	64969		368-16-1	906.3	168.08
		hydantoin-5-propionic acid	40473	C05565	HMDB01212	782		5624-26-0	2080	171.04
		imidazole propionate	40730		HMDB02271	70630		1074-59-5	2263	141.07
		imidazole lactate	15716	C05568	HMDB02320	440129		14403-45-3	2040	157.06
		1-methylimidazoleacetate	32350	C05828	HMDB02820	75810		2625-49-2	2064	141.07
		4-imidazoleacetate	32349	C02835	HMDB02024	96215		645-65-8	2236	125.04
	Lysine Metabolism	lysine	1301	C00047	HMDB00182	5962		56-87-1	2850	147.11
		N2-acetyllysine	36751	C12989	HMDB00446	92907		1946-82-3	3372.6	187.11
		N6-acetyllysine	36752	C02727	HMDB00206	92832		692-04-6	1059	187.11
		N6,N6,N6-trimethyllysine	1498	C03793	HMDB01325	440120		23284-33-5	2825	189.16
		5-hydroxylysine	15685	C16741	HMDB00450	1029		13204-98-3	2790	163.11
		2-aminoadipate	6146	C00956	HMDB00510	469		542-32-5;1118-90-7	3500	160.06
		glutarate (pentanedioate)	396	C00489	HMDB00661	743		110-94-1	2900	131.03
		glutarylcarnitine (C5)	44664		HMDB13130	71464488		102636-82-8	2393	276.14
		pipecolate	1444	C00408	HMDB00070	849		4043-87-2	2200	130.09
		6-oxopiperidine-2-carboxylate	43231			3014237		34622-39-4	1432	144.07
	Phenylalanine and Tyrosine Metabolism	phenylalanine	64	C00079	HMDB00159	6140		63-91-2	2878	166.09
		N-acetylphenylalanine	33950	C03519	HMDB00512	74839		2018-61-3	2597	206.08
		phenylpyruvate	566	C00166	HMDB00205	997		156-06-9	845	163.04
		phenyllactate (PLA)	22130	C05607	HMDB00779	3848		828-01-3	2208	165.06
		tyrosine	1299	C00082	HMDB00158	6057		60-18-4	2430	182.08
		N-acetyltyrosine	32390		HMDB00866	68310		537-55-3	1680	222.08

		4-hydroxyphenylpyruvate	1669	C01179	HMDB00707	979		156-39-8	1690.1	179.03
		3-(4-hydroxyphenyl)lactate	32197	C03672	HMDB00755	9378		6482-98-0	1379	181.05
		phenol sulfate	32553	C02180	HMDB60015	74426		937-34-8	2156	172.99
		p-cresol sulfate	36103	C01468	HMDB11635	4615423		3233-57-7	2890	187.01
		vanillylmandelate (VMA)	1567	C05584	HMDB00291	1245		55-10-7	941	197.05
		3-methoxytyrosine	12017		HMDB01434	1670		300-48-1	1606	210.08
		gentisate	18280	C00628	HMDB00152	3469		490-79-9	905	153.02
		3-phenylpropionate (hydrocinnamate)	15749	C05629	HMDB00764	107		501-52-0	2860	149.06
		thyroxine	2761	C01829	HMDB01918	5819		51-48-9	4808	775.68
		2-hydroxyphenylacetate	1432	C05852	HMDB00669	11970		614-75-5	858.6	151.04
		dopamine sulfate (2)	48407						1556.2	232.03
		p-cresol-glucuronide*	48841		HMDB11686	154035		17680-99-8	2420	283.08
		tyramine O-sulfate	48408		HMDB06409	153005		30223-92-8	1565	216.03
		5-bromotryptophan	53242			96735			3525	280.99
	Tryptophan Metabolism	tryptophan	54	C00078	HMDB00929	6305		73-22-3	2986	205.1
		N-acetyltryptophan	33959	C03137	HMDB13713	700653		1218-34-4	2630	245.09
		indolelactate	18349	C02043	HMDB00671	92904		832-97-3	2286	204.07
		indoleacetate	27513	C00954	HMDB00197	802		6505-45-9	2850	176.07
		indolepropionate	32405		HMDB02302	3744		830-96-6	3205	190.09
		3-indoxyl sulfate	27672		HMDB00682	10258		2642-37-7	2222.2	212
		kynurenine	15140	C00328	HMDB00684	161166		2922-83-0	2739	209.09
		kynurenate	1417	C01717	HMDB00715	3845		492-27-3	2224	188.04
		picolinate	1512	C10164	HMDB02243	1018		98-98-6	910	124.04
		5-hydroxyindoleacetate	437	C05635	HMDB00763	1826		54-16-0	1510.2	190.05
		serotonin	2342	C00780	HMDB00259	5202		153-98-0	2550	177.1
		tryptophan betaine	37097	C09213	HMDB61115	442106		20671-76-5	2673	247.14
		C-glycosyltryptophan	48782			10981970		180509-18-6	1777	365.14
		5-hydroxyindole sulfate	52914						1928	212

		thioproline	53231			93176;6973609		34592-47-4	1250	134.03
	Leucine, Isoleucine and Valine Metabolism	leucine	60	C00123	HMDB00687	6106		61-90-5	2864	132.1
		N-acetylleucine	1587	C02710	HMDB11756	70912		1188-21-2	2400	172.1
		4-methyl-2-oxopentanoate	22116	C00233	HMDB00695	70		816-66-0	2170	129.06
		isovalerylglycine	35107		HMDB00678	546304		16284-60-9	1950	158.08
		isovalerylcarnitine	34407		HMDB00688	6426851		31023-24-2	3085	246.17
		beta-hydroxyisovalerate	12129		HMDB00754	69362		625-08-1	1027	117.06
		3-methylglutaconate	38667		HMDB00522	1551553		5746-90-7	2579.6	143.03
		alpha-hydroxyisovalerate	33937		HMDB00407	99823		600-37-3	1126	117.06
		methylsuccinate	15745		HMDB01844	10349		498-21-5	2800	131.03
		isoleucine	1125	C00407	HMDB00172	6306		73-32-5	2800	132.1
		N-acetylisoleucine	33967			2802421		3077-46-1	2325	172.1
		3-methyl-2-oxobutyrate	44526	C00141	HMDB00019	49		3715-29-5	1465	115.04
		3-methyl-2-oxovalerate	15676	C00671	HMDB03736	47		1460-34-0;51829-07-3	2064.2	129.06
		2-methylbutyrylcarnitine (C5)	45095		HMDB00378	6426901		31023-25-3	3035	246.17
		2-hydroxy-3-methylvalerate	36746		HMDB00317	164623		488-15-3	1800	131.07
		3-hydroxy-2-ethylpropionate	32397		HMDB00396	188979		4374-62-3	975	117.06
		ethylmalonate	15765		HMDB00622	11756		601-75-2	2785	131.03
		valine	1649	C00183	HMDB00883	6287		72-18-4	2479	118.09
		N-acetylvaline	1591		HMDB11757	66789		96-81-1	1704	158.08
		isobutyrylcarnitine	33441		HMDB00736	168379		25518-49-4	2810	232.15
		isobutyrylglycine	35437		HMDB00730	10855600		15926-18-8	1420	144.07
		3-hydroxyisobutyrate	1549	C06001	HMDB00336	87		2068-83-9	1619	103.04
		alpha-hydroxyisocaproate	22132	C03264	HMDB00746	83697		10303-64-7	1840	131.07
	Methionine, Cysteine, SAM and Taurine	methionine	1302	C00073	HMDB00696	6137		63-68-3	2526	150.06
		N-acetylmethionine	1589	C02712	HMDB11745	448580		65-82-7	1787	190.05
		N-formylmethionine	2829	C03145	HMDB01015	439750		4289-98-9	1543.8	176.04

		methionine sulfone	44878			69961		820-10-0	1250	182.05
		methionine sulfoxide	18374	C02989	HMDB02005	158980		3226-65-1	1272	166.05
		S-adenosylhomocysteine (SAH)	42382	C00021	HMDB00939	439155		979-92-0	1832.4	383.11
		2-aminobutyrate	42374	C02261	HMDB00650	439691		1492-24-6	2059	104.07
		cysteine	1868	C00097	HMDB00574	5862		52-90-4;56-89-3	1488	122.03
		cystine	56	C00491	HMDB00192	67678		56-89-3	2000	241.03
		S-methylcysteine	39592		HMDB02108	24417		1187-84-4	880	134.03
		cysteine s-sulfate	22176	C05824	HMDB00731	115015		1637-71-4	565	201.98
		cysteine sulfinic acid	37443	C00606	HMDB00996	109		207121-48-0	597	154.02
		hypotaurine	590	C00519	HMDB00965	107812		300-84-5	724	110.03
		taurine	2125	C00245	HMDB00251	1123		107-35-7	690	124.01
		N-acetyltaurine	48187			159864			600	168.03
		2-hydroxybutyrate/2-hydroxyisobutyrate	52281						1258	103.04
	Urea cycle; Arginine and Proline Metabolism	arginine	1638	C00062	HMDB00517	232		1119-34-2	2825	175.12
		urea	1670	C00086	HMDB00294	1176		57-13-6	700	121.07
		ornithine	1493	C00077	HMDB03374	6262		3184-13-2	2800	133.1
		proline	1898	C00148	HMDB00162	145742		147-85-3	1603	116.07
		citrulline	2132	C00327	HMDB00904	9750		372-75-8	1520	176.1
		homoarginine	22137	C01924	HMDB00670	9085		156-86-5	2882	189.13
		homocitrulline	22138	C02427	HMDB00679	65072		1190-49-4	1908	190.12
		dimethylarginine (SDMA + ADMA)	36808	C03626	HMDB01539	123831		102783-24-4	2850	203.15
		N-acetylarginine	33953	C02562	HMDB04620	67427		155-84-0	2245	217.13
		N-acetylproline	34387			322640		1074-79-9	1695	158.08
		N-delta-acetylornithine	43249			9920500			858	173.09
		N-methylproline	37431			557		475-11-6	1335	130.09
		trans-4-hydroxyproline	32306	C01157	HMDB00725	5810		51-35-4	1064	132.07
		pro-hydroxy-pro	35127		HMDB06695	11673055		18684-24-7	2128	229.12

		N-monomethylarginine	43586	C03884	HMDB29416	132862		53308-83-1	2845	189.13
		N-acetylcitrulline	48434	C15532	HMDB00856	656979		33965-42-3	940	216.1
	Creatine Metabolism	creatine	27718	C00300	HMDB00064	586		57-00-1	1947	132.08
		guanidinoacetate	43802	C00581	HMDB00128	763		352-97-6	1937	118.06
	Polyamine Metabolism	acisoga	43258			129397		106692-36-8	1813	185.13
		spermidine	485	C00315	HMDB01257	1102		124-20-9	3355	146.17
		N(4)-acetylspermidine	32356			128317		66039-56-3	3095	188.18
		N-acetylputrescine	37496	C02714	HMDB02064	122356		18233-70-0	2230	131.12
		4-acetamidobutanoate	1558	C02946	HMDB03681	18189		3025-96-5	1350	146.08
	Guanidino and Acetamido Metabolism	1-methylguanidine	48114	C02294	HMDB01522	10111		22661-87-6	2149	74.07
		4-guanidinobutanoate	15681	C01035	HMDB03464	500		463-003;463-00-3	2320	146.09
		guanidinosuccinate	32446	C03139	HMDB03157	97856		6133-30-8	3550	174.05
	Glutathione Metabolism	5-oxoproline	1494	C01879	HMDB00267	7405		98-79-3	738.5	128.04
Peptide	Gamma-glutamyl Amino Acid	gamma-glutamylalanine	37063		HMDB29142	440103		5875-41-2	1986	219.1
		gamma-glutamylglutamate	36738	C05282	HMDB11737	92865		1116-22-9	1775	277.1
		gamma-glutamylglutamine	2730	C05283	HMDB11738	150914		10148-81-9	1430	276.12
		gamma-glutamylglycine	33949		HMDB11667	165527		1948-29-4	1535	205.08
		gamma-glutamylhistidine	18245			7017195		37460-15-4	2740	285.12
		gamma-glutamylisoleucine*	34456		HMDB11170	14253342			2940	261.14
		gamma-glutamylleucine	18369		HMDB11171	151023		2566-39-4	2991	261.14
		gamma-glutamyl-alpha-lysine	55015			65254			2784	276.16
		gamma-glutamyl-epsilon-lysine	33934		HMDB03869	7015684;7015685		17105-15-6	2717	276.16
		gamma-glutamylmethionine	44872		HMDB29155	7009567		17663-87-5	2640	279.1
		gamma-glutamylphenylalanine	33422		HMDB00594	111299		7432-24-8	2992	295.13

		gamma-glutamylthreonine	33364		HMDB29159			5652-48-2	1750	249.11
		gamma-glutamyltryptophan	33947		HMDB29160	3989307		66471-20-3	1960	332.13
		gamma-glutamyltyrosine	2734		HMDB11741	94340		7432-23-7	1240	309.11
		gamma-glutamylvaline	43829		HMDB11172	7015683		2746-34-1	2700	247.13
		gamma-glutamylserine	54914					5875-35-4	1351	235.09
	Dipeptide Derivative	N-acetylcarnosine	43488		HMDB12881	9903482		56353-15-2	2141	269.12
	Dipeptide	glycylvaline	18357		HMDB28854	97417		1963-21-9	2734	175.11
		histidylalanine	42027			351667		16874-75-2	3014	227.11
		leucylalanine	40010			259321		7298-84-2	2801	203.14
		leucylglycine	40045			79070		686-50-0	2778	189.12
		phenylalanylglycine	41370			98207		721-90-4	2462	221.09
		prolylglycine	40703			7408076;6426709		2578-57-6	2136	173.09
		threonylphenylalanine	31530			4099799;4099798		16875-27-7	2322	265.12
		valylglutamine	42079			5253209		42854-54-6	2295	246.14
		valylglycine	40475		HMDB29127	136487		686-43-1	1506	173.09
		valylleucine	39994		HMDB29131	352039		22906-55-4	3138	231.17
		leucylglutamine*	53061			4305457			1965	258.15
	Acetylated Peptides	phenylacetylglutamate	52925		HMDB59772	11579826			1630	264.09
		phenylacetylglutamine	35126	C04148	HMDB06344	92258		28047-15-6	2330	263.1
Carbohydrate	Glycolysis, Gluconeogenesis, and Pyruvate Metabolism	1,5-anhydroglucitol (1,5-AG)	20675	C07326	HMDB02712	64960		154-58-5	802	163.06
		glucose	48152	C00031	HMDB00122	79025		50-99-7	2342	225.06
		pyruvate	48990	C00022	HMDB00243	1060		127-17-3	1083	87.01
		lactate	527	C00186	HMDB00190	612		79-33-4	681.6	89.02
		glycerate	1572	C00258	HMDB00139	752		600-19-1	2070.4	105.02
	Pentose Metabolism	ribose	1471	C00121	HMDB00283	5779		50-69-1	1508.2	195.05
		ribitol	15772	C00474	HMDB00508	6912		488-81-3	1789.6	151.06

		ribonate	27731	C01685	HMDB00867	5460677		8/3/36	2425	165.04
		arabinose	575	C00216	HMDB00646	66308		28697-53-2	1804.5	195.05
		arabitol/xylitol	48885						1932.4	151.06
		ribulose/xylulose	54671						1400	195.05
		arabonate/xylonate	48255						2664.6	165.04
	Disaccharides and Oligosaccharides	sucrose	1519	C00089	HMDB00258	5988		57-50-1	3092	387.11
	Fructose, Mannose and Galactose Metabolism	fructose	48195	C00095	HMDB00660	5984		57-48-7	2022.2	225.06
		mannitol/sorbitol	46142	C01507	HMDB00247	5780			2260	181.07
		mannose	48153	C00159	HMDB00169	18950		3458-28-4	2200	225.06
		galactitol (dulcitol)	1117	C01697	HMDB00107	11850		608-66-2	2309.7	181.07
		galactonate	27719	C00880	HMDB00565	128869		299-28-5	3085	195.05
		glucuronate	15443	C00191	HMDB00127	444791		207300-70-7	3233.3	193.04
		N-acetylneuraminate	32377	C00270	HMDB00230	439197		131-48-6	660	310.11
	Aminosugar Metabolism	erythronate*	42420		HMDB00613	2781043		88759-55-1	2186	135.03
		N-acetylglucosamine/N-acetylgalactosamine	46539						715	222.1
	Advanced Glycation End-product	N6-carboxymethyllysine	36713					4/3/46	2500	205.12
Energy	TCA Cycle	citrate	1564	C00158	HMDB00094	311		77-92-9	582	191.02
		aconitate [cis or trans]	46173	C00417	HMDB00072				580	173.01
		isocitrate	12110	C00311	HMDB00193	1198		20226-99-7	773	210.06
		alpha-ketoglutarate	528	C00026	HMDB00208	51		305-72-6;328-50-7;22202-68-2	2700	145.01
		succinate	1437	C00042	HMDB00254	1110		110-15-6	3149	117.02

		fumarate	1643	C00122	HMDB00134	444972		100-17-8	3084	115
		malate	1303	C00149	HMDB00156	525		6915-15-7	615.6	133.01
		citraconate/glutaconate	47076						2600	129.02
		2-methylcitrate/homocitrate	52282						575	205.04
Lipid	Medium Chain Fatty Acid	caprate (10:0)	1642	C01571	HMDB00511	2969		112-37-8	5090.7	171.14
		undecanoate (11:0)	12067	C17715	HMDB00947	8180		334-48-5	5219.2	185.15
		10-undecenoate (11:1n1)	32497					1333-28-4	5085.8	183.14
		laurate (12:0)	1645	C02679	HMDB00638	3893		143-07-7	5300	199.17
		5-dodecenoate (12:1n7)	33968		HMDB00529	5312378		2430-94-6	5224	197.15
	Long Chain Fatty Acid	myristate (14:0)	1365	C06424	HMDB00806	11005		544-63-8	5440	227.2
		myristoleate (14:1n5)	32418	C08322	HMDB02000	5281119		544-64-9	5346.9	225.19
		pentadecanoate (15:0)	1361	C16537	HMDB00826	13849		1002-84-2;10002-84-2	5521	241.22
		palmitate (16:0)	1336	C00249	HMDB00220	985		57-10-3	5618	255.23
		palmitoleate (16:1n7)	33447	C08362	HMDB03229	445638		373-49-9	5475	253.22
		margarate (17:0)	1121		HMDB02259	10465		506-12-7	5731	269.25
		10-heptadecenoate (17:1n7)	33971		HMDB60038	5312435		29743-97-3	5555	267.23
		stearate (18:0)	1358	C01530	HMDB00827	5281		57-11-4	5872	283.26
		nonadecanoate (19:0)	1356	C16535	HMDB00772	12591		646-30-0	6068	297.28
		10-nonadecenoate (19:1n9)	33972		HMDB13622	5312513		73033-09-7	5780	295.26
		arachidate (20:0)	1118	C06425	HMDB02212	10467		506-30-9	6295	311.3
		eicosenoate (20:1)	33587		HMDB02231	5282768			5950	309.28
		erucate (22:1n9)	1552	C08316	HMDB02068	5281116		112-86-7	6355.6	337.31
		oleate/vaccenate (18:1)	52285						5655	281.25
		stearidonate (18:4n3)	33969	C16300	HMDB06547	5312508		111174-40-4	5395	275.2
		eicosapentaenoate (EPA; 20:5n3)	18467	C06428	HMDB01999	446284		10-2005-9;10417-94-4	5450	301.22
		docosapentaenoate (n3 DPA; 22:5n3)	32504	C16513	HMDB01976	6441454		2234-74-4	5571	329.25

		docosahexaenoate (DHA; 22:6n3)	44675	C06429	HMDB02183	445580		6217-54-5	5525	327.23
		linoleate (18:2n6)	1105	C01595	HMDB00673	5280450		60-33-3	5535	279.23
		linolenate [alpha or gamma; (18:3n3 or 6)]	34035	C06426	HMDB03073	5280934			5450	277.22
	Polyunsaturated Fatty Acid (n3 and n6)	dihomo-linolenate (20:3n3 or n6)	35718	C03242	HMDB02925	5280581		17046-59-2	5596	305.25
		arachidonate (20:4n6)	1110	C00219	HMDB01043	444899		506-32-1	5535	303.23
		adrenate (22:4n6)	32980	C16527	HMDB02226	5497181		2091-25-0	5678	331.26
		docosapentaenoate (n6 DPA; 22:5n6)	37478	C16513	HMDB01976	6441454		25182-74-5	5624.5	329.25
		docosadienoate (22:2n6)	32415	C16533	HMDB61714	5282807		7370-49-2	6034	335.3
		dihomo-linoleate (20:2n6)	17805	C16525	HMDB05060	6439848		2091-39-6	5730	307.26
		mead acid (20:3n9)	35174		HMDB10378	5312531		20590-32-3	5650	305.25
	Fatty Acid, Branched	13-methylmyristate	38293			151014		2485-71-4	5499	241.22
		15-methylpalmitate	38768			17903417			5695	269.25
		17-methylstearate	38296			3083779		2724-59-6	5993	297.28
	Fatty Acid, Dicarboxylate	3-methylglutarate/2-methylglutarate	54676						2832	145.05
		2-hydroxyglutarate	37253	C02630	HMDB00606	43		40951-21-1	3352.8	147.03
		adipate	21134	C06104	HMDB00448	196		124-04-9	3000	145.05
		3-methyladipate	36749		HMDB00555	12292		1/3/58	2865	159.07
		maleate	20676	C01384	HMDB00176	444266		110-16-7	2510	115
		pimelate (heptanedioate)	15704	C02656	HMDB00857	385		111-16-0	2745	159.07
		suberate (octanedioate)	15730	C08278	HMDB00893	10457		505-48-6	804.3	173.08
		azelate (nonanedioate)	18362	C08261	HMDB00784	2266		123-99-9	1276	187.1
		sebacate (decanedioate)	32398	C08277	HMDB00792	5192		111-20-6	1788	201.11
		dodecanedioate	32388	C02678	HMDB00623	12736		693-23-2	2990	229.14
		tetradecanedioate	35669		HMDB00872	13185		821-38-5	4000	257.18
		hexadecanedioate	35678	C19615	HMDB00672	10459		505-54-4	4615	285.21
		octadecanedioate	36754		HMDB00782	70095		871-70-5	5043	313.24
		eicosanodioate	39831			75502		2424-92-2	5200.9	341.27
		docosadioate	39837			244872		505-56-6	5291.8	369.3

		3-carboxy-4-methyl-5-propyl-2-furanpropanoate (CMPF)	31787		HMDB61112	123979		86879-39-2	2840	239.09
	Fatty Acid, Amino	2-aminoheptanoate	43761			227939		1115-90-8	3160	146.12
		2-aminooctanoate	43343		HMDB00991	69522		644-90-6	3385	158.12
	Fatty Acid, Keto	1-dihomo-linoleoylglycerol (20:2)	35103						6800	307.26
	Fatty Acid Synthesis	malonate	15872	C00383	HMDB00691	867		141-82-2;26522-22-85-0	3447	103
	Fatty Acid Metabolism (also BCAA Metabolism)	butyrylcarnitine	32412	C02862	HMDB02013	439829		25576-40-3	2860	232.15
		propionylcarnitine	32452	C03017	HMDB00824	107738		17298-37-2	2590	218.14
		propionylglycine	31932		HMDB00783	98681		21709-90-0	1502.5	130.05
		methylmalonate (MMA)	1496	C02170	HMDB00202	487		516-05-2	3078.4	117.02
	Fatty Acid Metabolism (Acyl Glycine)	N-palmitoylglycine	42092			151008		2441-41-0	5580	312.25
	Fatty Acid Metabolism(Acyl Carnitine)	acetylcarnitine	32198	C02571	HMDB00201	1		5080-50-2	2282	204.12
		3-hydroxybutyrylcarnitine (1)	43264		HMDB13127	53481617			2400	248.15
		hexanoylcarnitine	32328		HMDB00705	6426853		6920-35-0	3308	260.19
		octanoylcarnitine	33936	C02838	HMDB00791	123701		3671-77-0	950	288.22
		decanoylcarnitine	33941		HMDB00651	10245190		1492-27-9	1130	316.25
		cis-4-decenoyl carnitine	38178					98930-66-6	1057	314.23
		myristoylcarnitine	33952		HMDB05066	53477791		18822-89-4	1350	372.31
		palmitoylcarnitine	44681	C02990	HMDB00222	461		6865-14-1	1425	400.34
		palmitoleoylcarnitine*	53223						1357	398.33
		stearoylcarnitine	34409		HMDB00848	6426855		18822-91-8	1485	428.37
		linoleoylcarnitine*	46223		HMDB06469	6450015		36816-10-1	1430	424.34
		oleoylcarnitine	35160		HMDB05065	6441392;53477789		38677-66-6	1423	426.36

		myristoleoylcarnitine*	48182					889848-55-9	1316	370.29
	Carnitine Metabolism	deoxycarnitine	36747	C01181	HMDB01161	134		6249-56-5	2052	146.12
		carnitine	15500	C00318	HMDB00062	10917		461-05-2	1978	162.11
	Ketone Bodies	3-hydroxybutyrate (BHBA)	542	C01089	HMDB00357	441		625-72-9	1443.3	103.04
	Fatty Acid, Monohydroxy	2-hydroxyoctanoate	22036		HMDB02264	94180		617-73-2	3736.8	159.1
		2-hydroxydecanoate	42489			21488		5393-81-7	4840	187.13
		2-hydroxypalmitate	35675		HMDB31057	92836		764-67-0	5511.2	271.23
		2-hydroxystearate	17945	C03045		69417		629-22-1	5695	299.26
		3-hydroxyhexanoate	53230			151492		10191-24-9	1725	131.07
		3-hydroxyoctanoate	22001		HMDB01954	26613		88930-08-9	3446	159.1
		3-hydroxydecanoate	22053		HMDB02203	26612		5561-87-5	4634.7	187.13
		3-hydroxylaurate	32457		HMDB00387	94216		53941-38-1	5175	215.17
		16-hydroxypalmitate	39609	C18218	HMDB06294	10466		506-13-8	5236.2	271.23
		13-HODE + 9-HODE	37752			43013			5275	295.23
		3-hydroxystearate	52938			5282907		45261-96-9	5674	299.26
		2-hydroxylaurate	52916			97783		2984-55-6	5211	215.17
	Fatty Acid, Dihydroxy	12,13-DiHOME	38395	C14829	HMDB04705	10236635		263399-35-5	5137	313.24
		9,10-DiHOME	38399	C14828	HMDB04704	9966640		263399-34-4	5180	313.24
	Eicosanoid	leukotriene B4	37530	C02165	HMDB01085	5280492		71160-24-2	5186	335.22
		9-HETE	46301		HMDB10222	5312978			5341	319.23
	Endocannabinoid	oleoyl ethanolamide	38102		HMDB02088	5283454		11-58-0;111-58-0	6400	324.29
		palmitoyl ethanolamide	38165	C16512	HMDB02100	4671		544-31-0	6300	298.28

		linoleoyl ethanolamide	52608		HMDB12252	5283446		68171-52-8	6150	322.28
	Inositol Metabolism	myo-inositol	1124	C00137	HMDB00211	892		87-89-8	3506.3	225.06
	Phospholipid Metabolism	choline	15506	C00114	HMDB00097	305		67-48-1	1961	104.11
		choline phosphate	34396	C00588	HMDB01565	1014		72556-74-2	700	184.07
		glycerophosphorylcholine (GPC)	15990	C00670	HMDB00086	71920		28319-77-9	672	258.11
		glycerophosphoethanolamine	37455	C01233	HMDB00114	123874		33049-08-0	659	216.06
		trimethylamine N-oxide	40406	C01104	HMDB00925	1145		1184-78-7	2100	76.08
		glycerophosphoinositol*	47155					16824-65-0	3850	333.06
		1,2-dipalmitoyl-GPC (16:0/16:0)	19130		HMDB00564	452110		63-89-8	2365	734.57
		1-palmitoyl-2-oleoyl-GPC (16:0/18:1)	52461			6436017		26853-31-6;26853-31-6	2358	760.59
		1-palmitoyl-2-linoleoyl-GPC (16:0/18:2)	42446			5287971		40811-94-7	2160	758.57
		1-stearoyl-2-arachidonoyl-GPC (18:0/20:4)	42450			16219824		35418-59-8	2300	810.6
		1-stearoyl-2-oleoyl-GPC (18:0/18:1)	52438					56421-10-4	2644	788.62
		1-stearoyl-2-oleoyl-GPI (18:0/18:1)*	52726						3711	882.61
		1,2-dioleoyl-GPC (18:1/18:1)*	52457			10350317		4235-95-4	2346	786.6
		1-palmitoyl-2-arachidonoyl-GPC (16:0/20:4n6)	52462			10747814		35418-58-7	2091	782.57
		1-stearoyl-2-linoleoyl-GPC (18:0/18:2)*	52452						2380	786.6
		1-linoleoyl-2-linolenoyl-GPC (18:2/18:3)*	53176						1939	780.55
		1-palmitoyl-2-palmitoleoyl-GPC (16:0/16:1)*	52470						2160	732.55
		1-stearoyl-2-arachidonoyl-GPI (18:0/20:4)	52449						3000	904.59
		1-oleoyl-2-linoleoyl-GPC (18:1/18:2)*	52453						2165	784.59
		1-palmitoyl-2-linoleoyl-GPI (16:0/18:2)	52450						2730	852.56
		1-palmitoyl-2-arachidonoyl-GPI (16:0/20:4)*	52467						2567	876.56
		1-stearoyl-2-linoleoyl-GPI (18:0/18:2)	52468						3185	880.59
		1-palmitoyl-2-oleoyl-GPE (16:0/18:1)	19263		HMDB05320	5283496		26662-94-2	2509	718.54

		1-stearoyl-2-arachidonoyl-GPE (18:0/20:4)	52447			5289133			2424	768.55
		1-stearoyl-2-oleoyl-GPE (18:0/18:1)	42448						2858	746.57
		1-palmitoyl-2-arachidonoyl-GPE (16:0/20:4)*	52464		HMDB05323	9546800			2198	740.52
		1-palmitoyl-2-linoleoyl-GPE (16:0/18:2)	42449		HMDB05322	9546747			2275	716.52
		1-stearoyl-2-linoleoyl-GPE (18:0/18:2)*	52446			9546749			2522	744.55
		1-palmitoyl-2-stearoyl-GPC (16:0/18:0)	52616					59403-51-9	2653	762.6
		1,2-dioleoyl-GPE (18:1/18:1)	52609			9546757		5/1/04	2485	744.55
		1-palmitoyl-2-oleoyl-GPI (16:0/18:1)*	52669						3140	854.58
		1-palmitoyl-2-alpha-linolenoyl-GPC (16:0/18:3n3)*	52684						2034	756.55
		1-palmitoleoyl-2-linoleoyl-GPC (16:1/18:2)*	52683						2003	756.55
		1,2-dilinoleoyl-GPC (18:2/18:2)	52603			5288075		998-06-1	2006	782.57
		1,2-dilinoleoyl-GPE (18:2/18:2)*	53174			9546812			2083	740.52
		1-oleoyl-2-linoleoyl-GPE (18:1/18:2)*	52687		HMDB05349	9546753			2270	742.54
		1-linoleoyl-2-arachidonoyl-GPC (18:2/20:4n6)*	52710						1969	806.57
		arachidonoylcholine	53261						1390	390.34
		dihomo-linolenoyl-choline	53262						1424	392.35
		docosahexaenoylcholine	53263						1378	414.34
		oleoylcholine	53260						1449	368.35
		palmitoloelycholine	53257						1385	340.32
	Lysolipid	1-palmitoyl-GPC (16:0)	33955		HMDB10382	86554		17364-16-8	1525	496.34
		2-palmitoyl-GPC (16:0)*	35253		HMDB61702	15061532			1505	496.34
		1-palmitoleoyl-GPC (16:1)*	33230		HMDB10383	24779461			1450	494.32
		2-palmitoleoyl-GPC (16:1)*	35819						1425	494.32
		1-stearoyl-GPC (18:0)	33961		HMDB10384	497299		19420-57-6	1606	524.37
		1-oleoyl-GPC (18:1)	48258		HMDB02815	16081932		19420-56-5	1540	522.36
		1-linoleoyl-GPC (18:2)	34419	C04100	HMDB10386	11988421			1465	520.34
		1-linolenoyl-GPC (18:3)*	45951						1422	518.32
		1-arachidonoyl-GPC (20:4n6)*	33228	C05208	HMDB10395				1460	544.34
		1-palmitoyl-GPE (16:0)	35631		HMDB11503	9547069			1544	454.29
		1-stearoyl-GPE (18:0)	42398		HMDB11130	9547068		69747-55-3	1626	482.32
		2-stearoyl-GPE (18:0)*	41220						6350	480.31

		1-oleoyl-GPE (18:1)	35628		HMDB11506	9547071		89576-29-4	1554	480.31
		1-linoleoyl-GPE (18:2)*	36600		HMDB11507	52925130			1482	478.29
		1-arachidonoyl-GPE (20:4n6)*	35186		HMDB11517	42607465			1450	502.29
		1-palmitoyl-GPI (16:0)*	35305		HMDB61695				5564	571.29
		1-stearoyl-GPI (18:0)	19324		HMDB61696			796963-93-4	1700	601.33
		1-oleoyl-GPI (18:1)*	36602						5599	597.3
		1-linoleoyl-GPI (18:2)*	36594						5494	595.29
		1-arachidonoyl-GPI (20:4)*	34214		HMDB61690				5482	619.29
		1-palmitoyl-GPA (16:0)	34428	C04036	HMDB00327	6419701		17618-08-5	5600	409.24
		1-arachidonoyl-GPA (20:4)	46325					799268-65-8	5499	457.24
		1-oleoyl-GPA (18:1)	36812			5497152		325465-93-8	1635	437.27
		1-linoleoyl-GPA (18:2)*	52690		HMDB07856				5483	433.24
	Plasmalogen	1-(1-enyl-palmitoyl)-2-oleoyl-GPE (P-16:0/18:1)*	52477						2600	702.54
		1-(1-enyl-palmitoyl)-2-linoleoyl-GPE (P-16:0/18:2)*	52677						2351	700.53
		1-(1-enyl-palmitoyl)-2-palmitoyl-GPC (P-16:0/16:0)*	52716			11146967			2454	718.57
		1-(1-enyl-palmitoyl)-2-palmitoleoyl-GPC (P-16:0/16:1)*	52713						2218	716.56
		1-(1-enyl-palmitoyl)-2-arachidonoyl-GPE (P-16:0/20:4)*	52673						2270	724.53
		1-(1-enyl-palmitoyl)-2-oleoyl-GPC (P-16:0/18:1)*	52478						2443	744.59
		1-(1-enyl-stearoyl)-2-oleoyl-GPE (P-18:0/18:1)	52614					144371-68-6	2950	730.57
		1-(1-enyl-stearoyl)-2-linoleoyl-GPE (P-18:0/18:2)*	52748						2633	728.56
		1-(1-enyl-palmitoyl)-2-arachidonoyl-GPC (P-16:0/20:4)*	52689						2154	766.57
		1-(1-enyl-palmitoyl)-2-linoleoyl-GPC (P-16:0/18:2)*	52682						2226	742.57
		1-(1-enyl-stearoyl)-2-arachidonoyl-GPE (P-18:0/20:4)*	52475		HMDB05779	9547058			2511	752.56
	Lysoplasmalogen	1-(1-enyl-palmitoyl)-GPC (P-16:0)*	52474			10917802			1547	480.34
		1-(1-enyl-palmitoyl)-GPE (P-16:0)*	39270						1558	438.3
		1-(1-enyl-oleoyl)-GPE (P-18:1)*	44621						1566	464.31
		1-(1-enyl-stearoyl)-GPE (P-18:0)*	39271						1649	466.33
	Glycerolipid Metabolism	glycerol	15122	C00116	HMDB00131	753		56-81-5	758	91.04
		glycerol 3-phosphate	43847	C00093	HMDB00126	754		29849-82-9	580	173.02

		glycerophosphoglycerol	48857	C03274		439964			2430	245.04
	Monoacylglycerol	1-myristoylglycerol (14:0)	35625	C01885	HMDB11561	79050		75685-84-6	6353.3	227.2
		1-pentadecanoylglycerol (15:0)	47898			190750			6200	241.22
		1-palmitoylglycerol (16:0)	21127		HMDB31074	14900		542-44-9	6400	255.23
		2-palmitoylglycerol (16:0)	33419		HMDB11533	123409		23470-00-0	6300	255.23
		1-oleoylglycerol (18:1)	21184		HMDB11567	5283468		111-03-5	6500	281.25
		2-oleoylglycerol (18:1)	21232			5319879		3443-84-3	6300	281.25
		1-linoleoylglycerol (18:2)	27447			5283469		2277-28-3	6477	279.23
		2-linoleoylglycerol (18:2)	32506		HMDB11538	5365676		3443-82-1	6250	279.23
		1-linolenoylglycerol (18:3)	34393		HMDB11569	53480978			6214	277.22
		1-arachidonylglycerol (20:4)	34397	C13857	HMDB11572	5282281		35474-99-8	6450	303.23
		2-arachidonoylglycerol (20:4)	19266	C13856	HMDB04666	5282280		53847-30-6	6170	303.23
		1-docosahexaenoylglycerol (22:6)	35153		HMDB11587				6150	309.22
		1-dihomo-linolenylglycerol (20:3)	48341						6220	305.25
		1-palmitoleoylglycerol (16:1)*	52431						6094	253.22
	Diacylglycerol	diacylglycerol (16:1/18:2 [2], 16:0/18:3 [1])*	54966						2733	608.52
		oleoyl-linoleoyl-glycerol (18:1/18:2) [1]	46798					106292-55-1	3115	636.56
		oleoyl-linoleoyl-glycerol (18:1/18:2) [2]	46799					104346-53-4	3223	636.56
		oleoyl-linolenoyl-glycerol (18:1/18:3) [2]*	54970						2819	634.54
		oleoyl-arachidonoyl-glycerol (18:1/20:4) [2]*	54961						2990	660.56
		linoleoyl-arachidonoyl-glycerol (18:2/20:4) [1]*	54955						2566	658.54
		linoleoyl-arachidonoyl-glycerol (18:2/20:4) [2]*	54956						2624	658.54
		linoleoyl-linoleoyl-glycerol (18:2/18:2) [1]*	54968						2689	634.54
		linoleoyl-linolenoyl-glycerol (18:2/18:3) [1]*	54963						2426	632.52
		linoleoyl-linolenoyl-glycerol (18:2/18:3) [2]*	54964						2478	632.52
		palmitoyl-arachidonoyl-glycerol (16:0/20:4) [1]*	54957						2916	634.54
		palmitoyl-arachidonoyl-glycerol (16:0/20:4) [2]*	54958						3015	634.54
		palmitoleoyl-linoleoyl-glycerol (16:1/18:2) [1]*	54967						2664	608.52

		palmitoyl-oleoyl-glycerol (16:0/18:1) [1]*	54943						3562	612.56
		palmitoyl-oleoyl-glycerol (16:0/18:1) [2]*	54942						3695	612.56
		palmitoleoyl-oleoyl-glycerol (16:1/18:1) [1]*	52632		HMDB07131	9543694			3035	610.54
		palmitoleoyl-oleoyl-glycerol (16:1/18:1) [2]*	52631						3154	610.54
		palmitoyl-linoleoyl-glycerol (16:0/18:2) [1]*	52633		HMDB05207,HMDB07103	9543695			3080	610.54
		palmitoyl-linoleoyl-glycerol (16:0/18:2) [2]*	52634						3197	610.54
		palmitoyl-linolenoyl-glycerol (16:0/18:3) [2]*	54965						2834	608.52
		stearoyl-linoleoyl-glycerol (18:0/18:2) [2]*	54947						3762	638.57
		oleoyl-oleoyl-glycerol (18:1/18:1) [1]*	54945						3571	638.57
		oleoyl-oleoyl-glycerol (18:1/18:1) [2]*	54946						3695	638.57
		stearoyl-linoleoyl-glycerol (18:0/18:2) [1]*	54948						3650	638.57
	Sphingolipid Metabolism	sphinganine-1-phosphate	52605		HMDB01383	520		19794-97-9	1431	382.27
		N-palmitoyl-sphinganine (d18:0/16:0)	52604		HMDB11760	5283572		5966-29-0	3090	540.54
		N-behenoyl-sphingadienine (d18:2/22:0)*	57372						3911	620.6
		myristoyl dihydrosphingomyelin (d18:0/14:0)*	57365						2060	677.56
		palmitoyl dihydrosphingomyelin (d18:0/16:0)*	52434			9939965			2290	705.59
		behenoyl dihydrosphingomyelin (d18:0/22:0)*	57331						3150	789.68
		stearoyl sphingomyelin (d18:1/18:0)	19503	C00550	HMDB01348	6453725		85187-10-6;85187-10-6	2400	731.61
		tricosanoyl sphingomyelin (d18:1/23:0)*	52436						3200	801.68
		sphingomyelin (d18:1/14:0, d16:1/16:0)*	42463			11433862			1998	675.54
		sphingomyelin (d18:2/14:0, d18:1/14:1)*	47154						1860	673.53
		sphingomyelin (d18:1/15:0, d16:1/17:0)*	52433					121999-58-4	2082	689.56
		sphingomyelin (d18:2/16:0, d18:1/16:1)*	42459						2002	701.56
		sphingomyelin (d18:1/17:0, d17:1/18:0, d19:1/16:0)	52615					121999-64-2	2312	717.59
		sphingomyelin (d18:1/18:1, d18:2/18:0)	37529			6443882		108392-10-5	2167	729.59
		sphingomyelin (d18:1/20:0, d16:1/22:0)*	48490						2685	759.64
		sphingomyelin (d18:1/20:1, d18:2/20:0)*	48491					222403-67-0	2383	757.62
		sphingomyelin (d18:1/21:0, d17:1/22:0, d16:1/23:0)*	52495						2793	773.65

		sphingomyelin (d18:1/22:1, d18:2/22:0, d16:1/24:1)*	48493						2666	785.65
		sphingomyelin (d18:2/23:0, d18:1/23:1, d17:1/24:1)*	52435						2845	799.67
		sphingosine 1-phosphate	34445	C06124	HMDB00277	5283560		26993-30-6	1850	378.24
		N-palmitoyl-sphingosine (d18:1/16:0)	44877		HMDB04949	5283564		24696-26-2	2893	538.52
		N-stearoyl-sphingosine (d18:1/18:0)*	54979		HMDB04950	5283565		104404-17-3	3392	566.55
		glycosyl-N-palmitoyl-sphingosine	53013						2623	700.57
		glycosyl-N-stearoyl-sphingosine	52234						3053	728.6
		lactosyl-N-palmitoyl-sphingosine	53010					4201-62-1	2527	862.63
		lactosyl-N-nervonoyl-sphingosine (d18:1/24:1)*	57370						3778	972.73
	Mevalonate Metabolism	3-hydroxy-3-methylglutarate	531	C03761	HMDB00355	1662		503-49-1	2700	161.05
	Sterol	cholesterol	63	C00187	HMDB00067	11025495		57-88-5	2707	369.35
		7-alpha-hydroxy-3-oxo-4-cholestenoate (7-Hoca)	36776	C17337	HMDB12458	3081085		115538-85-7	5288.1	429.3
		3-hydroxy-5-cholestenoic acid	54805					6561-58-6	5398	415.32
		4-cholesten-3-one	38125	C00599	HMDB00921	91477		601-57-0	2520	385.35
	Steroid	pregnenolone sulfate	38170		HMDB00774	105074		1247-64-9	5100	395.19
		21-hydroxypregnenolone disulfate	46115	C05485	HMDB04026	134595		1164-98-3	3935	245.07
		5alpha-pregnan-3beta,20alpha-diol monosulfate (2)	37200						5060	399.22
		5alpha-pregnan-3beta,20alpha-diol disulfate	37198						3962	239.09
		pregnen-diol disulfate*	32562						3868	238.08
		pregn steroid monosulfate*	32619					1247-64-9	5000	397.21
		pregnanediol-3-glucuronide	40708			123796		1852-49-9	5145	495.3
		cortisol	1712	C00735	HMDB00063	5754		50-23-7	4713.2	361.2
		cortisone	1769	C00762	HMDB02802	222786		53-06-5	4572.5	359.19
		dehydroisoandrosterone sulfate (DHEA-S)	32425	C04555	HMDB01032	12594		651-48-9	4745	367.16
		16a-hydroxy DHEA 3-sulfate	38168						4240	383.15
		epiandrosterone sulfate	33973	C07635	HMDB00365			22229-22-7	4855	369.17

		androsterone sulfate	31591		HMDB02759	159663		2479-86-9	5022	369.17
		4-androsten-3beta,17beta-diol monosulfate (1)	37211		HMDB03818			521-17-5	4500	369.17
		4-androsten-3beta,17beta-diol monosulfate (2)	37210						4500	369.17
		4-androsten-3alpha,17alpha-diol monosulfate (2)	37207						4712	369.17
		4-androsten-3alpha,17alpha-diol monosulfate (3)	37209						5180	369.17
		4-androsten-3beta,17beta-diol disulfate (1)	37202	C04295	HMDB03818	10634			3740	224.06
		4-androsten-3beta,17beta-diol disulfate (2)	37203	C04295	HMDB03818	10634			4065	224.06
		5alpha-androstan-3alpha,17beta-diol monosulfate (1)	37186						4925	371.19
		5alpha-androstan-3beta,17beta-diol monosulfate (2)	37192						4685	371.19
		5alpha-androstan-3beta,17beta-diol disulfate	37190	C12525	HMDB00493	242332		571-20-0	3880	225.07
		andro steroid monosulfate (1)*	32827	C04555	HMDB02759				3871	383.15
		etiocholanolone glucuronide	47112					9/3/02	4915	465.25
	Primary Bile Acid Metabolism	cholate	22842	C00695	HMDB00619	221493		81-25-4	5165	407.28
		glycocholate	18476	C01921	HMDB00138	10140		475-31-0;863-57-0	5163	464.3
		chenodeoxycholate	1563	C02528	HMDB00518	10133		474-24-9;474-25-9	5264	391.29
		glycochenodeoxycholate	32346	C05466	HMDB00637	12544		16564-43-5	5236.1	448.31
		taurochenodeoxycholate	18494	C05465	HMDB00951	387316		6009-98-9	5250	498.29
	Secondary Bile Acid Metabolism	deoxycholate	1114	C04483	HMDB00626	222528		83-44-3	5294	391.29
		glycodeoxycholate	18477	C05464	HMDB00631	3035026		360-65-6	5281	448.31
		taurodeoxycholate	12261	C05463	HMDB00896	2733768		207737-97-1	5257.4	498.29
		glycolithocholate sulfate*	32620	C11301	HMDB02639	72222		15324-64-8	5011	255.63
		ursodeoxycholate	1605	C07880	HMDB00946	31401		128-13-2	5055	391.29
		glycoursodeoxycholate	39379		HMDB00708	12310288		64480-66-6	5033	448.31
		glycocholenate sulfate*	32599						4750	254.62
		taurocholenate sulfate	32807						4750	279.61
		glycodeoxycholate sulfate	52975						4875	263.63

	Fatty Acid Metabolism (Acyl Choline)	palmitoylcholine	52944			151731			1482	342.34
Nucleotide	Purine Metabolism, (Hypo)Xanthine/Inosine containing	inosine	1123	C00294	HMDB00195	6021		58-63-9	1602.1	267.07
		hypoxanthine	3127	C00262	HMDB00157	790		68-94-0	1291.2	135.03
		xanthine	3147	C00385	HMDB00292	1188		69-89-6	1032	153.04
		N1-methylinosine	48351		HMDB02721	65095		20245-33-4	1430	283.1
		urate	1604	C00366	HMDB00289	1175		69-93-2;120K5305	757.1	167.02
		allantoin	1107	C02350	HMDB00462	204		97-59-6	1672	157.04
	Purine Metabolism, Adenine containing	N1-methyladenosine	15650	C02494	HMDB03331	27476		15763-06-1	2120	282.12
		N6-carbamoylthreonyladenosine	35157		HMDB41623	161466		24719-82-2	2164	411.13
	Purine Metabolism, Guanine containing	7-methylguanine	35114	C02242	HMDB00897	11361		578-76-7	2175	166.07
	Pyrimidine Metabolism, Orotate containing	orotate	1505	C00295	HMDB00226	967		50887-69-9	1638.1	155.01
		orotidine	35172	C01103	HMDB00788	92751		314-50-1	2250	287.05
	Pyrimidine Metabolism, Uracil containing	uridine	606	C00299	HMDB00296	6029		58-96-8	1457.6	243.06
		pseudouridine	33442	C02067	HMDB00767	15047		1445-07-4	1100	243.06
		5-methyluridine (ribothymidine)	35136		HMDB00884	445408		1463-10-1	1778.1	257.08
		2'-deoxyuridine	52602	C00526	HMDB00012	13712		951-78-0	1586	227.07
		3-ureidopropionate	3155	C02642	HMDB00026	111		462-88-4	875	133.06
		beta-alanine	55	C00099	HMDB00056	239		56-41-7;107-95-9	1905	90.05
		N-acetyl-beta-alanine	37432	C01073		76406		3025-95-4	773	130.05
		5,6-dihydrothymine	1418	C00906	HMDB00079	93556		696-04-8	1280	129.07

	Pyrimidine Metabolism, Thymine containing	3-aminoisobutyrate	1566	C05145	HMDB03911	64956		10569-72-9;214139-20-5	2215	104.07
Cofactors and Vitamins	Nicotinate and Nicotinamide Metabolism	quinolinate	1899	C03722	HMDB00232	1066		89-00-9	3000	166.01
		nicotinamide	594	C00153	HMDB01406	936		98-92-0	1942	123.06
		1-methylnicotinamide	27665	C02918	HMDB00699	10129985		1005-24-9	1940	137.07
		trigonelline (N'-methylnicotinate)	32401	C01004	HMDB00875	5570		535-83-1	1388	138.05
		N1-Methyl-2-pyridone-5-carboxamide	40469	C05842	HMDB04193	69698		701-44-0	1250	153.07
	Pantothenate and CoA Metabolism	pantothenate	1508	C00864	HMDB00210	6613		137-08-6	1498.7	218.1
	Ascorbate and Aldarate Metabolism	threonate	27738	C01620	HMDB00943	151152		70753-61-6	2384	135.03
		oxalate (ethanedioate)	20694	C00209	HMDB02329	971		144-62-7	603	88.99
		gulonate*	46957		HMDB03290	9794176		20246-53-1	2750	195.05
	Tocopherol Metabolism	alpha-tocopherol	1561	C02477	HMDB01893	14985		59-02-9;10191-41-0	2522	430.38
		gamma-CEHC	44876		HMDB01931	133098		178167-75-4	3843	263.13
		gamma-tocopherol/beta-tocopherol	52473						2384	416.36
	Hemoglobin and Porphyrin Metabolism	bilirubin (Z,Z)	43807	C00486	HMDB00054	5280352		635-65-4	1840	585.27
		bilirubin (E,E)*	32586			5315454		114-25-0	4294	583.26
		biliverdin	2137	C00500	HMDB01008	5353439		55482-27-4;55482-27-4	1016	583.26
	Vitamin A Metabolism	retinol (Vitamin A)	1806	C00473	HMDB00305	445354		68-26-8	1636	269.23
	Vitamin B6 Metabolism	pyridoxate	31555	C00847	HMDB00017	6723		82-82-6	2185	182.05

Xenobiotics	Benzoate Metabolism	hippurate	15753	C01586	HMDB00714	464		495-69-2	2106.9	178.05
		2-hydroxyhippurate (salicylurate)	18281	C07588	HMDB00840	10253		487-54-7	2184	194.05
		3-hydroxyhippurate	39600		HMDB06116	450268		1637-75-8	1687	194.05
		4-hydroxyhippurate	35527		HMDB13678	151012		2482-25-9	1475	194.05
		benzoate	15778	C00180	HMDB01870	243		65-85-0	1750	121.03
		catechol sulfate	35320	C00090	HMDB59724	3083879		4918-96-1	1906	188.99
		O-methylcatechol sulfate	46111			22473			2344	203
		3-methyl catechol sulfate (1)	46165						2790	203
		4-methylcatechol sulfate	46146						2665	203
		4-ethylphenylsulfate	36099	C13637				123-07-9	3580	201.02
		4-vinylphenol sulfate	36098	C05627	HMDB04072	6426766		2628-17-3	3320	199.01
		3-methoxycatechol sulfate (1)	48763						2163	219
		methyl-4-hydroxybenzoate sulfate	48429						2873	231
	Xanthine Metabolism	caffeine	569	C07481	HMDB01847	2519		58-08-2	2106	195.09
		paraxanthine	18254	C13747	HMDB01860	4687		611-59-6	2279	179.06
		theobromine	18392	C07480	HMDB02825	5429		83-67-0	1600	181.07
		theophylline	18394	C07130	HMDB01889	2153		58-55-9	1870	181.07
		1-methylurate	34395	C16359	HMDB03099	69726		708-79-2	1900	181.04
		7-methylurate	39598			69160		612-37-3	1138	181.04
		1,3-dimethylurate	32391		HMDB01857	70346		944-73-0	1671.4	195.05
		1,7-dimethylurate	34400	C16356	HMDB11103	91611		33868-03-0	1578.6	195.05
		1,3,7-trimethylurate	34404	C16361	HMDB02123	79437		5415-44-1	1985	209.07
		1-methylxanthine	34389	C16358	HMDB10738	80220		6136-37-4	1568.8	165.04
		3-methylxanthine	32445	C16357	HMDB01886	70639		1076-22-8	1700	165.04
		7-methylxanthine	34390	C16353	HMDB01991	68374		552-62-5	1350	167.06
		5-acetylamino-6-amino-3-methyluracil	34424	C16366	HMDB04400	88299		19893-78-8	1710	197.07
	Food Component/ Plant	retinal	40807	C00376	HMDB01358,HMDB06218	638015			1666	285.22
		2,3-dihydroxyisovalerate	38276	C04039	HMDB12141	677		1756-18-9	1050	133.05
		2-isopropylmalate	15667	C02504	HMDB00402	77		3237-44-3	2605	175.06

		gluconate	587	C00257	HMDB00625	10690		527-07-1	2922	195.05
		cinnamoylglycine	38637		HMDB11621	709625		16534-24-0	3127	204.07
		dihydroferulic acid	40481			14340		1135-23-5	1928	195.07
		ergothioneine	37459	C05570	HMDB03045	3032311		58511-63-0	850	230.1
		erythritol	20699	C00503	HMDB02994	222285		149-32-6	1491	167.06
		homostachydrine*	33009	C08283	HMDB33433	441447		1195-94-4	1750	158.12
		methyl indole-3-acetate	1584		HMDB29738	74706		1912-33-0	3339	190.09
		N-(2-furoyl)glycine	31536		HMDB00439	21863		5657-19-2;5657-19-2	1525.8	168.03
		phytanate	598	C01607	HMDB00417,HMDB00801,HMDB00553	26840		14721-66-5	5908	311.3
		piperine	33935	C03882	HMDB29377	638024		94-62-2	1168	286.14
		quinate	18335	C00296	HMDB03072	6508		77-95-2	2432.9	191.06
		S-allylcysteine	43239			98280		21593-77-1	2690	162.06
		stachydrine	34384	C10172	HMDB04827	115244		4136-37-2	1440	144.1
		thymol sulfate	36095	C09908	HMDB01878			89-83-8	4410	229.05
		4-allylphenol sulfate	37181						3805	213.02
		methyl glucopyranoside (alpha + beta)	46144						1070	193.07
		pyrraline	48428					74509-14-1	2304	253.12
		2-keto-3-deoxy-gluconate	48141		HMDB01353	161227		17510-99-5	1900	177.04
	Bacterial/ Fungal	tartronate (hydroxymalonate)	20693	C02287	HMDB35227	45		80-69-3	610	119
	Drug	hydroquinone sulfate	35322	C00530	HMDB02434	161220		123-31-9	1395	188.99
		salicylate	1515	C00805	HMDB01895	338		69-72-7	802	137.02
	Chemical	2-pyrrolidinone	31675		HMDB02039	12025		616-45-5	1262	86.06
		sulfate*	46960	C00059	HMDB01448	1118		14808-79-8	616	96.96
		O-sulfo-L-tyrosine	45413			514186			990	260.02

		2-aminophenol sulfate	43266		HMDB61116	181670			1677	188
		dimethyl sulfone	43424	C11142	HMDB04983	6213		67-71-0	832	95.02
		ectoine	35651	C06231		126041		96702-03-3	1980	143.08
		lanthionine	42002			6994972;98504		8/2/83	1730	209.06
		succinimide	41888	C07273		11439		123-56-8	2155	116.04
		N-methylpipecolate	47101			11862129;11286529		41447-17-0	1932	144.1
		4-hydroxychlorothalonil	48441			34217		28343-61-5	4427	244.91
		1,2,3-benzenetriol sulfate (2)	48762						1709	204.98
		3-hydroxypyridine sulfate	48448					1955-23-3	1595.5	173.99
		6-hydroxyindole sulfate	48698						2498	212

Table S2. Fold Changes of Metabolites in Refined Profile

Fold Change: Resistant$_{WTC-LI}$/Control

<1

>1

Super Pathway	Sub Pathway	Metabolite	Comp ID	KEGG	HMDB	PubChem	Resistant$_{WTC-LI}$ / Control	CAS	Retention Index	Mass
Amino Acid	Glycine, Serine and Threonine Metabolism	Serine	1648	C00065	HMDB00187	5951		56-45-1	1239	106.05
	Phenylalanine and Tyrosine Metabolism	N-acetylphenylalanine	33950	C03519	HMDB00512	74839		2018-61-3	2597	206.08
	Tryptophan Metabolism	N-acetyltryptophan	33959	C03137	HMDB13713	700653		1218-34-4	2630	245.09
		Serotonin	2342	C00780	HMDB00259	5202		153-98-0	2550	177.1
	Leucine, Isoleucine and Valine Metabolism	N-acetylleucine	1587	C02710	HMDB11756	70912		1188-21-2	2400	172.1
		Isovalerylglycine	35107		HMDB00678	546304		16284-60-9	1950	158.08
		N-acetylisoleucine	33967			2802421		3077-46-1	2325	172.1
		Isobutyrylglycine	35437		HMDB00730	10855600		15926-18-8	1420	144.07
	Methionine, Cysteine, SAM and Taurine Metabolism	N-formylmethionine	2829	C03145	HMDB01015	439750		4289-98-9	1543.8	176.04
	Urea cycle; Arginine and Proline Metabolism	Arginine	1638	C00062	HMDB00517	232		1119-34-2	2825	175.12
		Citrulline	2132	C00327	HMDB00904	9750		372-75-8	1520	176.1
Carbohydrate	Pentose Metabolism	Arabinose	575	C00216	HMDB00646	66308		28697-53-2	1804.5	195.05
		Ribulose/xylulose	54671						1400	195.05

Energy	TCA Cycle	Malate	1303	C00149	HMDB00156	525		6915-15-7	615.6	133.01
Lipid	Polyunsaturated Fatty Acid (n3 and n6)	Eicosapentaenoate (EPA; 20:5n3)	18467	C06428	HMDB01999	446284		10-2005-9;10417-94-4	5450	301.22
	Fatty Acid Metabolism (also BCAA Metabolism)	Methylmalonate (MMA)	1496	C02170	HMDB00202	487		516-05-2	3078.4	117.02
	Fatty Acid Metabolism(Acyl Glycine)	N-palmitoylglycine	42092			151008		2441-41-0	5580	312.25
	Fatty Acid, Monohydroxy	2-hydroxypalmitate	35675		HMDB31057	92836		764-67-0	5511.2	271.23
		2-hydroxystearate	17945	C03045		69417		629-22-1	5695	299.26
		2-hydroxylaurate	52916			97783		2984-55-6	5211	215.17
	Phospholipid Metabolism	1-stearoyl-2-arachidonoyl-GPC (18:0/20:4)	42450			16219824		35418-59-8	2300	810.6
		1-linoleoyl-2-arachidonoyl-GPC (18:2/20:4n6)*	52710						1969	806.57
		Dihomo-linolenoyl-choline	53262						1424	392.35
		Oleoylcholine	53260						1449	368.35
	Lysolipid	2-palmitoyl-GPC (16:0)*	35253		HMDB61702	15061532			1505	496.34
	Sterol	7-alpha-hydroxy-3-oxo-4-cholestenoate (7-Hoca)	36776	C17337	HMDB12458	3081085		115538-85-7	5288.1	429.3
	Primary Bile Acid Metabolism	Chenodeoxycholate	1563	C02528	HMDB00518	10133		474-24-9;474-25-9	5264	391.29

Nucleotide	Pyrimidine Metabolism, Orotate containing	Orotate	1505	C00295	HMDB00226	967		50887-69-9	1638.1	155.01
Xenobiotics	Xanthine Metabolism	Theobromine	18392	C07480	HMDB02825	5429		83-67-0	1600	181.07
	Food Component/Plant	Phytanate	598	C01607	HMDB00417, HMDB00801, HMDB00553	26840		14721-66-5	5908	311.3
